# Progressive neuronal plasticity in primate visual cortex during stimulus familiarization

**DOI:** 10.1126/sciadv.ade4648

**Published:** 2023-03-24

**Authors:** Kenji W. Koyano, Elena M. Esch, Julie J. Hong, Elena N. Waidmann, Haitao Wu, David A. Leopold

**Affiliations:** ^1^ Section on Cognitive Neurophysiology and Imaging, National Institute of Mental Health, Bethesda, MD 20892, USA.; ^2^ Chemistry and Synthesis Center, National Heart, Lung and Blood Institute, National Institutes of Health, Bethesda, MD 20892, USA.; ^3^ Neurophysiology Imaging Facility, National Institute of Mental Health, National Institute of Neurological Disorders and Stroke, National Eye Institute, Bethesda MD 20892, USA.

## Abstract

The primate brain is equipped to learn and remember newly encountered visual stimuli such as faces and objects. In the macaque inferior temporal (IT) cortex, neurons mark the familiarity of a visual stimulus through response modification, often involving a decrease in spiking rate. Here, we investigate the emergence of this neural plasticity by longitudinally tracking IT neurons during several weeks of familiarization with face images. We found that most neurons in the anterior medial (AM) face patch exhibited a gradual decline in their late-phase visual responses to multiple stimuli. Individual neurons varied from days to weeks in their rates of plasticity, with time constants determined by the number of days of exposure rather than the cumulative number of presentations. We postulate that the sequential recruitment of neurons with experience-modified responses may provide an internal and graded measure of familiarity strength, which is a key mnemonic component of visual recognition.

## Teaser

Sequential neural plasticity in the IT cortex marks the graded familiarity of learned stimuli.

## INTRODUCTION

The neocortex of the mammalian brain processes complex sensory information (*1*) and stores memory for subsequent recognition across specialized circuits (*2*, *3*). Primates have an advanced capacity for the visual comprehension of objects and social stimuli, which is reflected in specialized visual regions of the inferior temporal (IT) cortex (*4*, *5*). Neurons in IT are known to exhibit experience-dependent response plasticity following repeated exposure to the same visual stimulus (*6*–*8*). This plasticity is often manifest as diminished responses to familiar visual objects compared to novel ones, particularly in the late phase of the response (*9*–*14*). At the same time, methodological restrictions have limited our mechanistic understanding of visual response plasticity, including its temporal dynamics and its coordinated emergence within a neural population. What is the time frame for the establishment and persistence of neuronal plasticity? Is the rate of plasticity governed principally by the accumulation of exposures to a given visual stimulus? Do neighboring neurons in a local population exhibit experience-dependent changes in concert? The answers to these and related questions are unknown because it is difficult to longitudinally record the activity of single neurons across multiple sessions, particularly from deep structures in the brain.

**Fig. 1.**
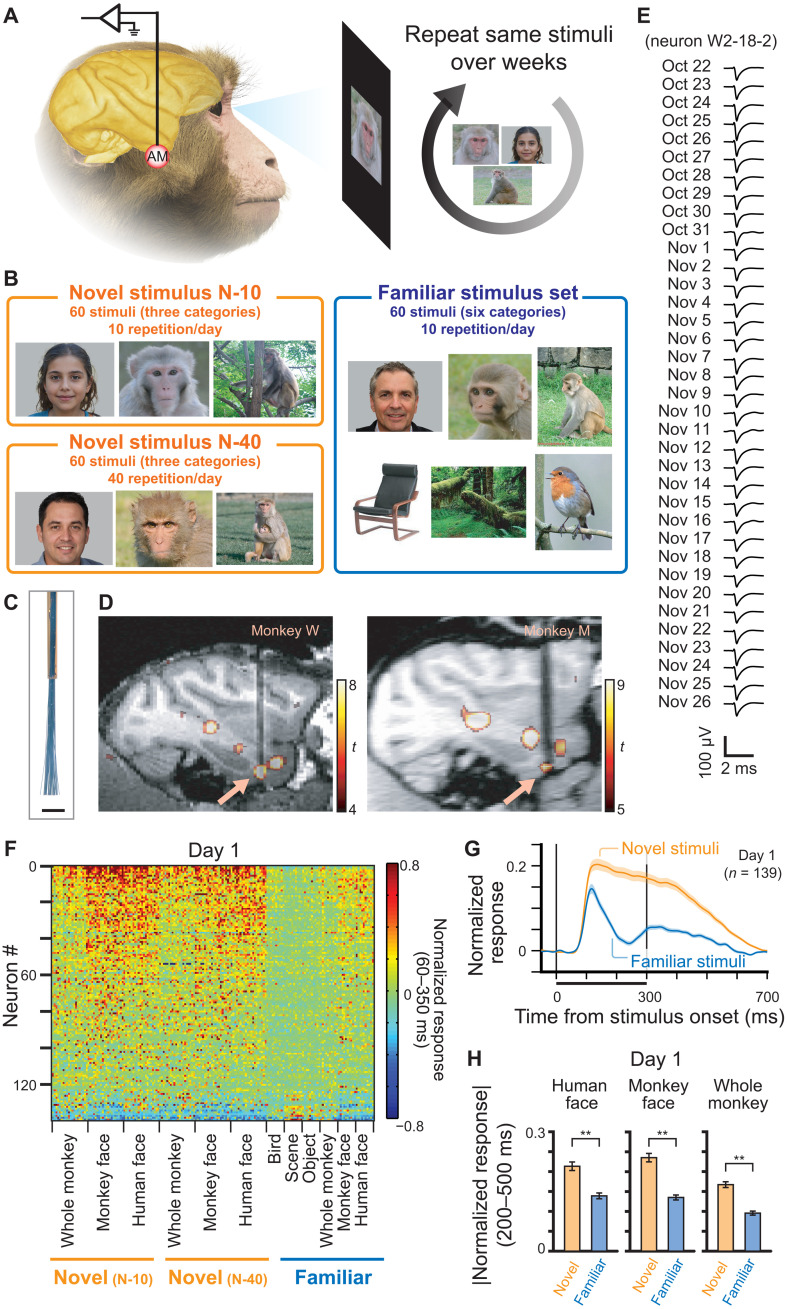
Experimental design and population response to the stimuli. (**A**) Overview of the experiment. Neurons were recorded from AM face patch, while stimuli were repeatedly shown to the monkeys over the course of 2 to 5 weeks. (**B**) Three categories (novel N-10, novel N-40, and familiar) of visual stimuli. (**C**) Electrode tip of the microwire brush array. Scale bar, 1 mm. (**D**) Recording location of each monkey, shown in sagittal section of MRI. Arrows indicate AM face patch. (**E**) Example waveform of a neuron recorded over 36 days. (**F**) Response of recorded neurons to each stimulus on the first day of the recording. Neurons were sorted according to the response to face stimuli compared to other stimuli. (**G**) Time course of responses to novel and familiar stimuli (human face, monkey face, and whole monkey) on the first day. Shaded area indicates standard error from mean (SE). (**H**) Average response to each category of stimulus on the first day during the late sustained response. Ordinate, absolute value of normalized responses. Error bars indicate SE. **P < 10^−14^, paired *t* test; *t*_138_ = 8.95 (human face), 9.79 (monkey face), and 11.85 (whole monkey). Because of privacy right reasons, we display mock stimulus images for human faces in A and B, which were generated by artificial intelligence (https://this-person-does-not-exist.com/en).

Here, we examined the unfolding of neural plasticity in a face-selective region of the IT cortex of the macaque by tracking responses of isolated single neurons for periods of up to 5 weeks of daily exposure to visual stimuli. During these periods, we randomly interleaved presentations of 120 visual stimuli that were initially unfamiliar to the animals and 60 stimuli that were highly familiar from the first presentation. Longitudinal tracking with a flexible microwire electrode array allowed us to observe how electrophysiological responses of neurons gradually signaled the memory for individual stimuli across recording days (Fig. 1, A and B). We report that during periods of familiarization with new stimuli, neurons’ late-phase spiking responses to a given stimulus diminished gradually, with time constants of days to weeks. Moreover, neighboring neurons in a local population exhibited highly distinct characteristic time constants for plasticity. Control experiments revealed that these time constants depended primarily on the number of exposure days to a given stimulus, rather than the accumulated number of presentations. We discuss the potential bearing of the observed plasticity on the familiarity component of visual recognition.

## RESULTS

We used flexible microwire brush array electrodes to record single neuron activity longitudinally over weeks. Before any visual exposure to the stimuli, the electrodes were surgically implanted into the functional magnetic resonance imaging (fMRI)–defined anterior medial (AM) face patch (Fig. 1, A, C, and D). The AM face patch is the most anteroventral node in the macaque IT face processing network, sitting adjacent to the perirhinal cortex, and is thought to play an important role in processing facial identity (*15*). The microwires permitted isolation and tracking of the same neurons during the experimental sessions over weeks (Fig. 1E and fig. S1) (*16*–*18*). We carried out longitudinal recordings from the AM face patch from two animals through four extended experimental sessions, each ranging from 11 to 37 days of continuous recording. Throughout these sessions, we isolated a total of 4874 neural waveforms over 90 days of recordings (54.8 ± 5.4 isolated neurons/day). We restricted analysis to 139 neurons that were longitudinally recorded between 7 and 37 days (fig. S2). These neurons were responsive to at least one stimulus (*P* < 0.05, *t* test versus baseline response with Bonferroni correction). Of 139 neurons, 117 (84.2%) were face selective (face-selective index > 0.33; see Materials and Methods and fig. S3).

Consistent with previous reports, population responses on the first experimental day were larger to novel stimuli than to familiar stimuli (Fig. 1F), and this difference was expressed primarily during the late sustained response (Fig. 1G and see figs. S4 and S5) (*10*–*14*). Similar patterns of late-phase response suppression were observed for familiar images of human faces, monkey faces, and whole monkeys (Fig. 1H and fig. S6).

**Fig. 2.**
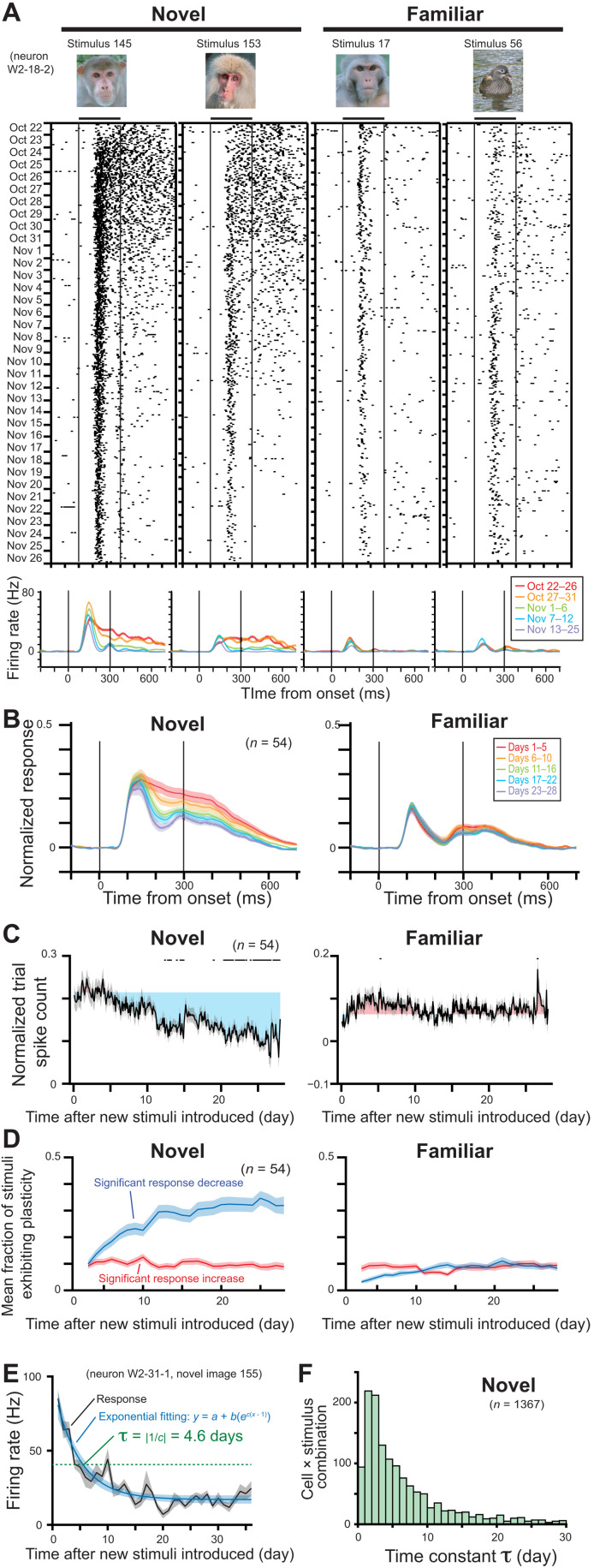
Decrease of late, sustained responses over multiple days of visual exposure to initially novel stimuli. (**A**) Response of an example neuron over five weeks of recording, showing adapting responses to novel (left) but not familiar (right) stimuli. (**B**) Population-averaged spike density functions for novel (N-10) and familiar stimuli. Responses are normalized with response to the best stimulus and baseline activity. (**C**) Change of response during late sustained period of 200 to 500 ms from stimulus onset over 4 weeks. All presentation trials are plotted in order, with days indicated at bottom. Black dots on the top indicate trials whose responses are significantly different from the responses of first 2 days (paired *t* test, *P* < 0.05 with Bonferroni correction). Gray shaded area indicates SE from mean. Colored shaded area indicates difference from mean response of first 2 days. (**D**) Mean fraction of stimuli showing significant population response changes from first 2 days (*t* test, *P* < 0.05). (**E**) An example of exponential fitting and computation of τ for response changes for a given neuron × stimulus combination across the extended recording session. (**F**) Distribution of time constants τ for all neuron × stimulus combinations in the novel (N-10) stimulus set.

Figure 2A shows the responses of an example neuron to four stimuli recorded daily over a period of 5 weeks, starting on October 22. Two of the stimuli were initially novel and then presented 10 times/day. The other two were highly familiar (presented 685 times through 48 days; see Materials and Methods) and also presented 10 times/day. The novel stimuli at first elicited sustained spike trains, lasting well beyond the removal of the stimulus (696 and 602 ms from stimulus onset for image 145 and 153, respectively; see also fig. S7). However, these sustained responses decreased very gradually, over the course of several days, until they were no longer detectable after 2.5 weeks, around November 10 (Fig. 2A, left two columns). The early visual response between 100 and 200 ms after the stimulus onset was comparatively unchanged during this period and still evident on November 26, after 5 weeks of exposure to the novel stimuli (see also figs. S8, C and G, and S9 for slight increase of the early visual response in some cases). Responses of the same neuron to familiar stimuli were much weaker relative to the novel stimuli beginning on the first experimental day, a finding that was representative across the population (Fig. 1, F to H), and relatively constant through the 5 weeks (Fig. 2A, right two columns).

To examine these response changes across the population of neurons, we analyzed recording sessions that were conducted over a period of greater than 4 weeks in each monkey. Across a population of 54 neurons recorded at least 20 of the first 28 days (mean recording days during the first 28 days = 26.0 ± 2.5 days), a gradual decline in late-phase responses was evident (Fig. 2, B and C, and see also fig. S8 for other example neurons and figs. S10 to S12 for additional quantifications). Analysis revealed that the sustained responses to novel stimuli decreased significantly (*P* < 10^−6^, *t*_46_ = 5.85, paired *t* test between first and last 2 days; Fig. 2, B and C, left), while the corresponding responses to familiar stimuli did not (*P* = 0.25, paired *t* test, *t*_46_ = 1.16; Fig. 2, B and C, right). The fraction of novel stimuli whose population responses were affected by this form of experience-dependent plasticity grew to reach 31.9 ± 3.3% after 4 weeks (Fig. 2D, left, mean ± SE; see also figs. S13 and S14). In contrast, the fraction of the affected familiar stimuli was minimal (Fig. 2D, right). While most changes were expressed during the sustained period, early transient responses did show a small response increase for a subset of novel stimuli (11.4 ± 1.6% at 4 weeks later, mean ± SE; fig. S9). The observed response change could not be attributed to nonspecific factors, such as a general adaptation of the cell or a loss of spike isolation over time, because a new set of novel stimuli introduced in the middle of the experimental session initially elicited strong sustained responses, which subsequently declined over time (fig. S15). The response change tended to be larger for stimuli that elicited stronger responses (figs. S12, S14, S16, and S17), maintaining a relatively unchanging stimulus selectivity and response sharpness over the course of visual exposures (figs. S16 and S17).

To quantify the time constant of the response decay following the introduction of novel stimuli, we fit the responses of each neuron to each stimulus with an exponential function (Fig. 2E and see also figs. S18 to S20), matching the observed response decay and eventual attainment of a stable value. We restricted our analysis to responses from 1367 neuron × stimulus combinations that could be fit well with an exponential through the course of the session of 11 to 36 days (see Materials and Methods for detailed criteria). As only a subset of stimuli showed this behavior for a given neuron, this criterion limited analysis to 16.4% of all possible neuron × stimulus combinations. Consistent with the observed decrease in mean response (Fig. 2C), these fits also showed that the polarity of the change was mostly decreasing (fig. S21). We defined the time constant τ as the time point when the neuron’s response decreased to 1/*e*, or 36.8%, of the response on the first day (green dotted line in Fig. 2E). Across the 1367 combinations, the distribution of τ had a long tail, ranging from 2 days to nearly 30 days. The median τ value was 4.23 days (Fig. 2F), after which responses are estimated to decrease by 95% after 12.7 days. This range of time constants for stimulus familiarity is broadly consistent with estimations from previous studies tracking multi-unit responses (*19*) and responses of different neurons (*20*) across days. There was no clear relationship between the time constant and magnitude of the response change (fig. S22). Quantifying the time constant of individual IT neurons enabled us to further evaluate important factors for the time course and population dynamics as described in the following sections.

**Fig. 3.**
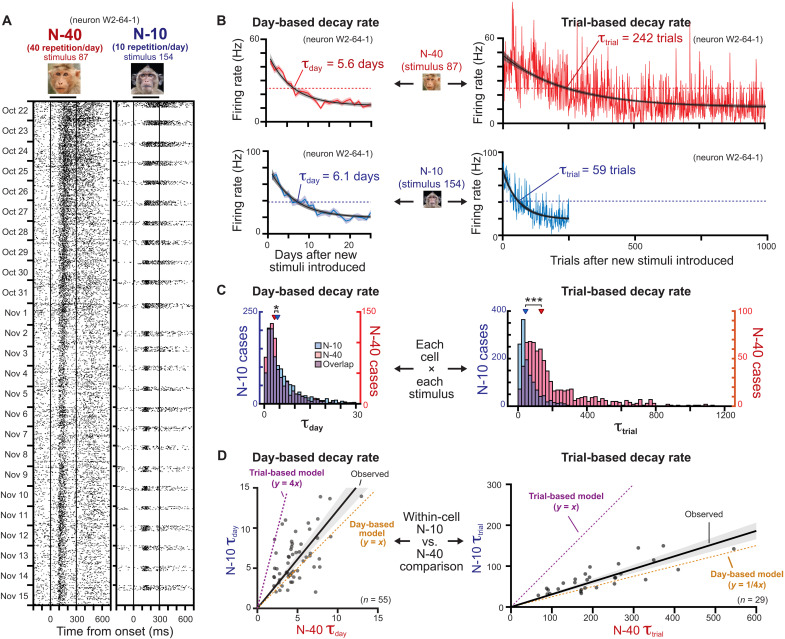
Primary factor of day on the rate of visual response plasticity. (**A**) Response change of an example neuron for one of N-40 and N-10 stimuli. (**B**) Exponential fitting used to calculate day-based (left) and trial-based (right) decay rates. Top two plots: Example for the N-40 stimulus. Bottom two plots: Example for the N-10 stimulus. The good correspondence between day-based decay rates and divergent trial-based decay rates for the N-40 and N-10 stimuli indicate that day is the critical factor. (**C**) Population distribution of estimated decay rates for each cell × stimulus combination. Left: Day-based decay rate (τ_day_). Right: Trial-based decay rate (τ_trial_). *P < 10^−5^ and ***P < 10^−122^, Mann-Whitney *U* test. (**D**) Within-cell comparison between N-10 and N-40 stimuli. Each dot represents a cell. The τ values of each cell are the mean of all the stimuli with valid exponential fit. The black regression line is calculated from observed τ with a linear regression model *y* = *ax*.

The response to novel stimuli decreased with repeated daily exposure to the same stimuli. Would these changes proceed faster if more repetitions were presented each day? We tested this question by conducting an additional longitudinal experiment in both animals comparing the time constant between two novel stimulus sets, one shown 10 times/day (N-10 stimulus set) and the other shown 40 times/day (N-40 stimulus set). Figure 3A shows the responses of an example cell for two stimuli, including one from N-10 and one from the N-40 stimulus sets. Both sets of stimuli show gradually decreasing sustained responses over weeks. To weigh the relative contribution of total days versus total number of presentations, we computed both time- and trial-based exponential models for the N-10 and N-40 stimuli (Fig. 3B). This analysis revealed that the number of days determined the rate of response change to a much higher degree than did the number of presentations. In this example, this can be seen in the similar day-based time constant (τ_day_; Fig. 3B, left) but strongly divergent trial-based time constant (τ_trial_; Fig. 3B, right).

Across the population, the number of days, rather than the accrued number of stimulus presentations, was the critical determinant of response adaptation rate (Fig. 3C). For the N-40 and N-10 stimulus sets, the overall distributions of τ_day_ for all neuron × stimulus combinations was highly similar, although there was a small difference (median 3.31 versus 4.24 days; *P* < 10^−6^, Mann-Whitney *U* test). In contrast, the median distributions for τ_trial_ differed approximately by a factor of 4 (median 135.2 versus 42.7 trials; *P* < 10^−121^, Mann-Whitney *U* test).

Comparing the mean time constants of individual neurons of both animals for the N-10 and N-40 stimuli similarly indicated that day was the critical variable (Fig. 3D and see also fig. S11C). Neurons had very similar τ_day_ values for the two conditions and were thus distributed near the unity line (Fig. 3D, left, black regression line, slope of 1.22); however, τ_trial_ values differed by approximately nearly a factor of 4 (Fig. 3D, right, black regression line, slope of 0.31). We also confirmed that day was the critical factor from the beginning of the visual exposure and later period (fig. S23). These results indicate that the number of elapsed days during periods of visual exposure critically determines the rate of response plasticity among single neurons.

Most of the analysis thus far considered the temporal dynamics of plasticity regarding all possible neuron × stimulus combinations. Given the broad distribution of well-modeled time constants, we next asked whether the rate of plasticity is set by individual neurons or by individual stimuli. For example, if each neuron has a unique and fixed time constant, then a given neuron should show the same rate of plastic changes for multiple different stimuli. On the other hand, if the rate of plasticity is determined principally by details of the stimulus, then the entire neural population should adjust its activity in concert. In that case, a given neuron could exhibit vastly different plasticity time constants for different stimuli.

**Fig. 4.**
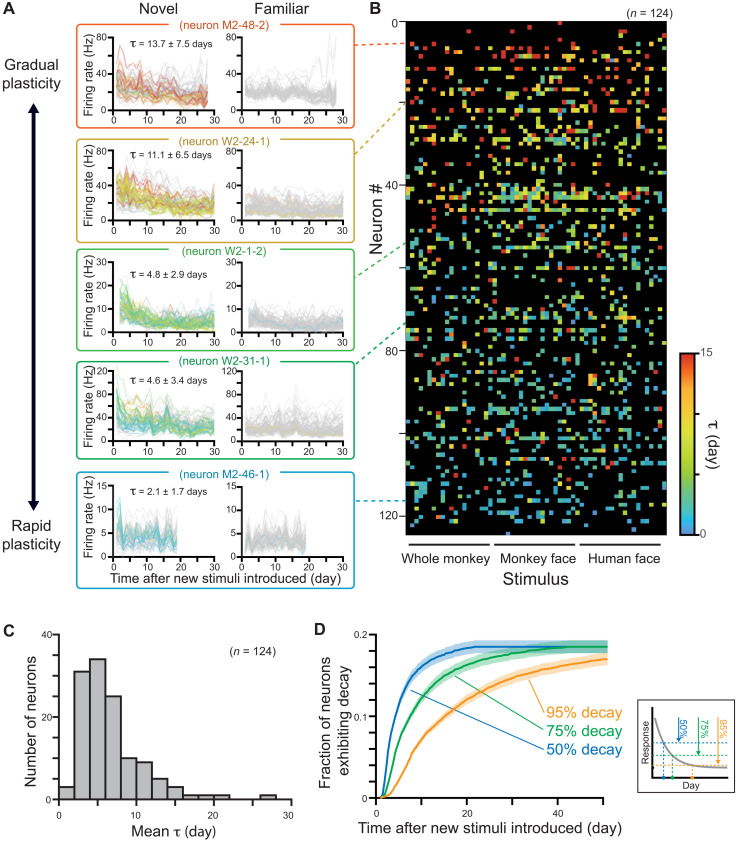
Progressive neuronal plasticity during the repeated presentation of stimuli across weeks. (**A**) Example neurons highlighting the wide range of plasticity time constants across the population. The colors indicate the τ_day_ for each stimulus depicted in the color scale in (B). Gray traces correspond to stimuli that did not elicit significant plasticity. Neurons were sorted with average time constant, from gradual plasticity neurons (top) to rapid plasticity neurons (bottom). The average time constant is calculated from the colored traces which exhibit change of responses and fulfills criteria of fitting quality. (**B**) Time constant for all cell × stimulus combinations across the population, showing dependence of the length of time constant on neuron’s identity. Black squares correspond to stimuli that do not exhibit change of responses or do not meet the criteria of fitting quality. Neurons had similar time constants for multiple stimuli, whereas individual stimuli did not show shared time constants across neurons (see also figs. S24 to S26). (**C**) Distribution of mean τ of each neuron, calculated from the stimuli, which exhibit change of responses and fulfills criteria of fitting quality. (**D**) Ratio of neurons exceeding 50, 75, and 95% decay threshold over days, which is estimated from τ of each neuron × stimulus combination. The fraction of neurons is calculated for each stimulus and then averaged. Shaded area represents SE. Inset: Definition of time when a neuron exceeds decay thresholds.

We thus analyzed the plasticity time constants as a function of both neurons and stimuli (Fig. 4). We found that neural identity had a much stronger role in determining the rate of plasticity than did stimulus identity. For individual cells, the plasticity rate was comparable for different stimuli; however, for a given stimulus, the plasticity rate varied widely across different neurons (Fig. 4A and see also fig. S24B for high variance of plasticity rate for individual stimulus). Across the population of the 1367 neuron × stimulus combinations, cell identity had a very strong and significant effect on the time constant (*P* < 10^−33^, Kruskal-Wallis test; Fig. 4B and see also figs. S11D, S19C, and S24 to S26). The main effect of cell identity was robust and reproduced in each experimental session (*P* < 0.02 to *P* < 10^−8^, fig. S24; *P* < 0.0005 to *P* < 10^−16^, fig. S25) and for the N-40 stimuli (*P* < 10^−29^). The main effect of stimulus identity was weaker and significant only in some recording sessions (figs. S24 to S26).

These results indicate that the population of neurons in the AM face patch has a broad range of unique cell-specific time constants governing their plasticity. These time constants ranged from 2 days to more than 20 days (Fig. 4C and see also fig. S19B). Following the introduction of new stimuli, the number of neurons showing modified responses to the stimuli thus increases over time. Figure 4D shows that, depending on the criterion for response modification, the proportion of participating neurons grows gradually over days and weeks. In the case of the AM face patch, nearly 20% of the recorded neurons, on average, eventually exhibited plasticity to the face and body stimuli we showed.

## DISCUSSION

The observation that individual IT neurons have unique time constants for visual plasticity suggests a division of labor that may bear on the graded nature of familiarity in visual recognition (*21*, *22*). The brain’s internal signal for stimulus familiarity is thought to be continuous and to increase with increasing exposure. Our findings suggest that visual face-selective neurons may contribute to this internal signal through a sequential progression of neuronal commitment to the familiarity of a stimulus. According to this hypothesis, the most rapidly adapting neurons we observed are specialized to identify and remember new experiences, perhaps opening the door to the recruitment of additional neurons following further exposure. With increasing experience playing out across days, neurons with longer plasticity time constants join the subpopulation of affected neurons. Over time, this progression of neural plasticity across the population creates an internal and gradually intensifying stimulus-specific signal of familiarity that could serve visual recognition.

One might attribute the diminishing of responses over time to waning attention, as monkeys are known to prefer looking at novel stimuli, similar to humans (*23*, *24*). However, the wide range of observed plasticity time constants, spanning from 2 to 30 days, makes any straightforward explanation involving a time course of attention seem unlikely. Moreover, an analysis of pupillometry failed to detect significant change of attention-related pupil dilation over the stimulus exposure period (fig. S27, A and B). Notwithstanding, familiarity and attention are mutually related psychological factors, as seen in the animal’s natural preference to novel objects. The strength of familiarity might drive animal’s attention. Although it is difficult to quantify the intensity of subjectivefamiliarity, especially in animals (fig. S27C), the development of improved psychological measures may enhance understanding of the specific relationship between fading attention and strengthening familiarity, as well as the neural contribution of the two factors.

The psychological variable of familiarity is most frequently associated with the perirhinal cortex (*9*, *20*, *25*–*28*) and has recently been associated with an adjacent region in the temporal pole (*29*). Nonetheless, the evidence for involvement of IT cortex in this process is also strong (*9*–*14*). For example, IT neurons are more selective (*11*, *14*, *30*, *31*), respond more reliably (*13*) to, and show sharper dynamic responses (*12*) for familiar stimuli, suggesting that experience has the capacity to shape high-level sensory processing in favor of known stimuli. Reduced responses to familiar visual stimuli were also reported in the prefrontal (*32*, *33*) and earlier visual areas (*19*), suggesting involvement of brain-wide network to represent the effect of familiarity. Given the delayed onset of the familiarity signal during the late sustained response observed in this and previous (*14*, *19*) studies, familiarity is likely to be represented through inter-areal interaction, such as the back propagation signals from higher areas (*34*). Future studies using simultaneous recording from multiple areas of the brain would further elucidate the mechanism of the plasticity network across the whole brain.

The face patch AM, from which we recorded, is directly adjacent to the perirhinal cortex and is most commonly associated with the encoding of individual face identity (*15*, *35*). In AM and several other lateral and ventral face patches, Landi and Freiwald (*28*) found diminished fMRI activation for visually familiar versus nonfamiliar faces. By contrast, face patches far away from this region, such as those in the superior temporal sulcus (STS), showed weak, if any, effects of familiarity. Perhaps for this reason, an earlier longitudinal recording from the anterior fundus (AF) face patch showed little evidence of response changes over several months (*16*, *17*). Face patches in the STS fundus region may be principally concerned with dynamic aspects of facial behavior and may therefore offer less contribution to visual recognition (*36*–*38*). Whether attending to changing aspects of faces, such as motion, gaze, and expression contributes to long-term plasticity in such areas is a question for the future. Future studies examining the neural expression of familiarity in other IT subregions can clarify whether the plasticity observed here reflects the position of the AM face patch or generalizes to other IT locations.

A potentially unexpected finding was that the rate of plasticity was determined principally by the number of exposure days, rather than by the cumulative number of stimulus exposures. This finding suggests that there may be an upper limit on the number of stimulus exposures that can contribute to plasticity in a given day, which resonates with the known importance of rest and sleep after the learning to consolidate the acquired memory (*2*). The slow development of response change over days might reflect accumulation of learned information, discretized over a nightly process of consolidation.

Primates are highly social animals that form large groups and interact competitively with other groups. Accurately learning and retaining knowledge about individuals, including their facial structure and behavior, is a critical aspect of daily survival. Our findings that AM neurons in the adult brain exhibit progressive plasticity for faces may tie directly to primates’ lifelong capacity to learn new individuals or to improve their capacity to read and interpret the faces of their conspecifics. It is notable that the plasticity observed in the present study was manifested during the late-phase response, similar to the late-phase responses involved in analyzing the details of facial identity (*39*) or in discounting average facial structure in theories of norm-based encoding (*40*, *41*). It is possible that plasticity of late-phase responses in the AM face patch additionally serves the continual updating of an internal model of facial statistics to aid in identifying individuals or interpreting facial behavior. Previous studies reported sharpening of response selectivity to unfamiliar objects in IT neurons with visual exposure (*11*, *14*, *30*, *31*). In contrast, we found that the neurons in AM face patch keep their stimulus selectivity constant during the development of the familiarity signal. This discrepancy may be partly because this study had not designed to evaluate change of parameters within the face feature space and partly because of a unique property of face-selective neurons that have pre-existing specialization. Future studies specifically designed to investigate face identity learning using parameterized face stimuli may elucidate changes in facial encoding such as axis coding (*42*) and norm-based coding (*40*). Similar to the familiarity signal, this process may benefit from the gradual commitment of neurons to identity dimensions over visual experience and may thus share overlapping neural mechanisms with the gradual familiarization of novel stimuli studied here. For example, during the learning process, rapidly adapting neurons can quickly modulate their tuning for new identities, amid a larger population of more stable neurons that maintains codes for reliable recognition. The mixture of stable and flexible information may be a universal property of adaptable systems in the brain, as similar principles appear to be present in other structures, such as the basal ganglia (*43*) and hippocampus (*44*).

## MATERIALS AND METHODS

### Subjects

Two rhesus monkeys (*Macaca mulatta*, both male, monkey W and M weighing 8.5 and 9.3 kg, respectively) were used in this study. All animals were surgically implanted with an MRI-compatible head post and with a chronic microwire electrode bundle in an AM face patch (Fig. 1D), which was functionally localized using a standard fMRI block design using movie clips (*45*, *46*) and/or a naturalistic movie watching paradigm (*47*). The apparatus and surgical implantation protocol have been described in detail previously (*17*). All surgeries were performed under aseptic conditions and general anesthesia under isoflurane, and animals were given postsurgical analgesics and prophylactic antibiotics. During participation in the recording experiment, the animals were on water control and received their daily fluid intake during their testing (see below). The weight and hydration level of each subject were monitored closely and maintained throughout the experimental testing phases. All the experimental procedures and animal welfare were in full compliance with the *Guidelines for the Care and Use of Laboratory Animals* by U.S. National Institutes of Health and approved by the Animal Care and Use Committee of the U.S. National Institutes of Mental Health/National Institutes of Health.

### Behavioral task and visual stimuli

The animals were not required to perform a behavioral task that required cognitive efforts for recognition of identities. With the passive viewing paradigm, neurons were examined for sensory processing of visual face stimuli. The monkeys sat in a primate chair in front of a light emitting diode (LED)/organic LED (OLED) monitor with their head position stabilized by means of an implanted head post. They were required to maintain their gaze on a fixation point of 0.2° × 0.2° at the center of the monitor through a trial. In each trial, visual stimuli of 15° diagonal length (7.3° to 12.9° × 7.6° to 13.1°) were presented for 300 ms in pseudo-random order followed by a 400-ms interstimulus interval. The monkeys were rewarded with fruit juice for successfully maintaining fixation within a window of 1.5° to 2°, while their eye position was monitored using an infrared tracking system (EyeLink II, SR Research). Pupil diameter is also monitored and collected in two of the experimental series (series W-1 and W-2; fig. S27). Stimulus presentation, eye position monitoring, and reward delivery were controlled by MonkeyLogic software (*48*) or NIMH MonkeyLogic Software (*49*). The monitor (either a ViewSonic 18″ LCD monitor or LG 55″ OLED monitor, both 60-Hz refresh rate) was placed 90 cm in front of the monkey. The timing of stimulus presentation was recorded by a photodiode sensor that received signal from a small white square displayed on a corner of the screen at the same time of stimulus presentation.

The main stimulus set comprised three groups of stimuli, each of which included 60 images (Fig. 1B). One stimulus set was highly familiar to the animals, and the other two stimulus sets were novel to the animals at the beginning of each series of experimental session. The familiar stimulus set includes images from six categories: human face, monkey face, whole monkey, object, scene, and bird (10 images from each category). Each of the two novel stimulus sets includes images from three categories: human face, monkey face, and whole monkey (20 images from each category). The human face images were drawn from the FEI face database (*50*), and monkey faces and bodies were provided courtesy of O. Dal Monte. Images from all other stimulus categories were assembled from Web searches or iPhone applications (*17*). Because of privacy right reasons, we display mock stimulus images for human faces in Fig. 1 and figs. S1, S8, S15, and S24, which were generated by artificial intelligence (https://this-person-does-not-exist.com/en). The original stimulus images used in the experiments can be provided upon request. The images of the familiar stimulus set had been used to confirm neurons’ consistent responses across days (also see the “Electrophysiology” section below) in other studies and repeatedly presented to the animals before starting the first data collection of this study. For monkey M, the familiar stimuli had been presented 68,948 times in total (mean: 1149 times per each stimulus) through 65 experimental days before the first data collection of this study. For monkey W, the familiar stimuli had been presented 41,102 times in total (mean: 685 times per each stimulus) through 48 experimental days before the first data collection of this study. We continued to use the same familiar images during data collection in this study. The images of the novel stimulus sets had never been shown to the animals until starting data collection for this study, and we prepared new stimuli when starting new series of experimental session. The same novel stimuli were used for both animals (One novel stimulus set was used in experimental series W-1 and M-1, and another novel stimulus set was used in experimental series W-2 and M-2; see also fig. S2). The familiar stimulus set and one of the novel stimulus sets (N-10) were shown to the animals 10 times/day, while the other novel stimulus set (N-40) was shown 40 times/day. In addition to the three major stimulus sets described above, another “intermittent” novel stimulus set was also shown to the animals 10 times on the first day and on the 11th or 12th day. In one series of experimental session (session W-2), this intermittent stimulus set was also shown to the animal on days 19, 26, 33, 36, and 37. The detailed stimulus presentation schedule is shown in fig. S2. There are total of four series of experimental sessions, each of which continued for 11, 11, 28, and 37 days, respectively. In two longer series of experimental sessions, (sessions M-2 and W-2), additional new novel stimulus sets (N-10_2_, N-10_3_, and N-10_4_) were introduced at the middle of the experimental sessions on days 13, 19, and 26. These additional novel stimulus sets were shown 10 times a day. To keep the total number of image presentation per day the same, 15 images were randomly dropped from N-40 stimulus set each time when the new stimulus set was introduced. In one of the recording sessions (W-1), familiar and intermittent stimulus sets were presented 1 day before and 1 day after the series of the experimental session only because the animal could not complete larger number of trials during that time.

### Electrophysiology

Extracellular neuronal signals were recorded with 64 chronically implanted NiCr wires (Microprobes; Fig. 1C) that permitted tracking of individual neurons over multiple recording sessions (*16*–*18*). The recorded neuronal signals were amplified and digitized at 24.4 kHz in a radio frequency–shielded room by PZ5 NeuroDigitizer (Tucker-Davis Technologies) and then stored to an RS4 Data Streamer controlled by an RZ2 BioAmp Processor (Tucker-Davis Technologies). A gold wire inserted into a skull screw was used for ground. Broadband signals (2.5 to 8 kHz) were collected from which individual spikes were extracted offline using the WaveClus software (*51*) after filtering between 300 and 5000 Hz. Of the 139 units we analyzed, most of the units (131, 94.2%) had broad spikes that exhibited spike trough-to-peak time longer than 0.8 ms. Considering that neurons with broad spikes constitute ~85% of the neurons in the cortex, the higher ratio of broad spike neurons in this study suggests potential bias of electrophysiological isolation by the brush array electrodes toward broad spike neurons (fig. S17). Event codes, eye positions, and a photodiode signal were also stored to a hard disk using OpenEX software (Tucker Davis Technologies).

The method for longitudinal identification of neurons across days was described in detail previously (*16*, *17*). The spikes recorded from the same channel on different days routinely had closely matching waveforms and interspike interval histograms and were provisionally inferred to arise from the same neurons across days. This initial classification based purely on waveform features and spike statistics was further tested against the pattern of stimulus selectivity and temporal structure of the neurons’ firing evoked by visual stimulation with the familiar stimulus set (fig. S1). We used the distinctive visual response pattern to the familiar stimuli generated by isolated spikes as a neural “fingerprint” to further disambiguate the identity of single units over time (*40*, *52*). The stability of stimulus selectivity was assessed by calculating a correlation coefficient for two firing rate vectors for the 60 familiar stimuli between two consecutive days (fig. S1, D and G). In addition, the consistency was confirmed by the stability of firing rate strength during the baseline period (−100 to +50 ms relative to stimulus onset; fig. S1E) and mean response to the familiar stimuli (60 to 350 ms from stimulus onset; fig. S1G). For subsequent analysis we used 139 neurons which were isolated at least for 7 days (fig. S2). Some neurons were isolated throughout a series of recording sessions, while some other neurons disappear, emerge and reappear during the recording session.

### Quantification and statistical analysis

Stored neuronal response data were analyzed offline with MATLAB software (MathWorks, MA). All the data in the text were expressed as means ± SD unless otherwise stated. Error bars in figures are SE unless otherwise stated. All the *t* test statistics were two-tailed. When a neuron was not isolated on a given day and the data are missing, we perform statistics without the data (Fig. 2B, seven neurons were not isolated on the first or last two days and excluded from the *t* test; fig. S1E, three neurons were not isolated on the first or last 2 days in session W-1 and excluded from the *t* test). Firing rate responses of neurons to each stimulus were calculated for the following periods: baseline period, 150 ms before the stimulus onset to 50 ms after stimulus onset; response period, 60 to 350 ms after the stimulus onset; late sustained period, 200 to 500 ms after the stimulus onset; and early transient response period, 50 to 150 ms after the stimulus onset. Significant responses to each stimulus were evaluated by *t* test with Bonferroni correction for firing rate responses between the baseline and response period. All the 139 neurons showed significant response to at least one stimulus of the 180 novel or familiar stimuli and were considered as responsive to the stimuli. Population-averaged tuning was calculated by averaging normalized firing rate response, which was calculated from the maximum and baseline responses of each neuron. Maximum response was defined as the response to the stimulus that elicited the largest response during the response period, either excitation or suppression, as compared to the baseline response. The response for each stimulus was normalized by subtracting the baseline response and then dividing it by the absolute difference between the baseline and maximum response of the neuron, resulting in normalized firing rate value that ranges from −1 to 1 (−1 or 1 corresponded to the maximum response and 0 corresponded to the baseline response). Neurons whose mean response was less than the baseline were considered as suppressive neurons (*n* = 13, 9.4%), and the sign of their normalized firing rates was inverted before calculating the population-averaged response. Spike trains were smoothed by convolution with a Gaussian kernel (σ = 10 ms) to obtain spike density functions (SDFs) for each stimulus. SDF was normalized as the normalized firing rate response by subtracting the baseline response and then dividing it by the absolute difference between the baseline and maximum response of the neuron. Face selectivity index (FSI) (*53*, *54*) was calculated from the mean baseline-subtracted responses to familiar faces (*X*_faces_) and familiar nonface images (*X*_nonface_) as: *FSI* = (*X*_faces_ – *X*_nonface_)/(|*X*_faces_| + |*X*_nonface_|). In case *FSI* > 1, *FSI* = 1. In case *FSI* < −1, *FSI* = −1. *FSI* = −*FSI* when both *X*_faces_ and *X*_nonface_ are negative to incorporate inhibitory face-selective response. *FSI* varies between −1 and 1. When the *FSI* of a neuron was larger than 0.33, the neuron was considered as face selective. Selectivity index (*55*) was calculated from number of stimuli k and response to *i*-th stimuli *i_n_* as: Selectivity index = [*k*−(Σ_*n*=1,*k*_ *i_n_*/*i*_max_)]/(*k* – 1). Selectivity (*14*, *30*) was Michelson contrast between the responses to the best and worst stimuli, which was calculated as: (Response_best_ – Response_worst_)/(Response_bset_ + Response_worst_). Sparseness (*11*) was calculated from number of stimuli *k* and response to *i*-th stimuli *i_n_* as: Sparseness = (1 – *A*)/(1 – 1/*k*), where *A* = (Σ_*n*=1,*k*_ *i_n_*/*k*)^2^/Σ_*n*=1,*k*_ (*i_n_*^2^/*k*). Kurtosis was calculated as a measure of tuning sharpness (*56*, *57*).

Time constants of response changes of each neuron were calculated for each stimulus by fitting the responses over the series of experimental session with an exponential function: *y* = *a* + *b · e*^*c*(*x*−1)^, where *x* is the day after new stimuli introduced, *y* is firing rate, and *e* is Euler’s number. We used exponential function because the response change is expected to reach a stable value at some time point, and it actually fit well with many of the changing responses (e.g. Fig. 2E). The optimal modeling parameters for *a*, *b*, and *c* were estimated by least squares technique with a maximum of 2000 iterations, within a predefined limited range to avoid overfitting: [−500 to 500] for *a*, [−1000 to 1000] for *b*, and [−10 to 10] for *c*. Then, we defined the time constant τ as |1/*c*|, which corresponds to the time point when the neuron’s response decreased to 1/*e*, or 36.8%, of the response on the first day.

For the population-averaged plot of normalized firing rate, we performed the analysis for neurons, which was recorded at least for 70% of total length of evaluated period. For the longer recording sessions of both animals (sessions M-2 and W-2; fig. S2), 54 (81.8%) out of 66 neurons fulfilled this criterion for the first 28 days of recording (Fig. 2, B to D). We limited our analysis of the time constant τ only for good fitting of exponential function: Exponential fitting was evaluated if the coefficient of determination (*R*^2^) value of the fitting is larger than 0.3 (42.3% of all fitting), if the neuron is recorded at least 7 days during first 10 days (95.0% of all fitting), and if the neuron’s response firing rate to the stimulus is larger than 2 Hz (78.4% of all fitting). In addition to the criteria above, we applied two more criteria to evaluated τ only when the neuron’s response changes during the experimental period: We evaluated τ if the neuron’s firing rate is significantly different between first and last 2 days by *t* test (*P* < 0.05, 24.9% of all fitting) and if τ is smaller than 30 days. The second criteria were applied to exclude fitting with very long τ that change the response very slowly [this excluded 254 (3%) cases whose τ was 884.7 ± 637.7 days] and thus difficult to be evaluated from our experiment of less than 40 days. Fitting with negative τ values, which do not reach plateau response, was also excluded (10 cases, 0.1%). With the criteria above, 1367 (16.4%) fitting of 8340 neuron × stimulus combinations (139 neurons × 60 stimuli) were considered (Figs. 2, E and F; 3C; and 4). Of 139 neurons, 124 (89.2%) have at least one fitting to a stimulus and was considered (Fig. 4B). For the time constant τ_trial_, *R*^2^ value threshold of 0.05 was used instead of 0.3, because the data are not averaged each day and thus noisier than that of τ_day_. For Fig. 3D, if the neurons have at least 10 stimuli, which fulfilled the above criteria of exponential regression in both N-10 and N-40 stimulus sets, τ values were averaged across stimuli and used for the analysis of the scatter plot (*n* = 56 for τ_day_ and *n* = 32 for τ_trial_).

Kruskal-Wallis test was performed to evaluate the effect of cell identity on τ values. To quantify the effect of cell identity and stimulus identity on τ values at the same time, τ values were log-transformed and then two-way analysis of variance (ANOVA) was applied with cell identities and stimulus identities as main factors. Interaction between the two factors was not incorporated in the ANOVA model because each of stimulus × cell combination had a single τ value. Explained variances by each factor were calculated using *R*^2^ statistics from the ANOVA table.
