# Supplementary material for: Progressive neuronal plasticity in primate visual cortex during stimulus familiarization
Source: Sci Adv. 2023 Mar 24;9(12):eade4648. doi: 10.1126/sciadv.ade4648 (PMC10038346; doi:10.1126/sciadv.ade4648)
Supplement: Supplementary file 1 — Figs. S1 to S27 [file sciadv.ade4648_sm.pdf]

Science Advances
AAAS

Supplementary Materials for

Progressive neuronal plasticity in primate visual cortex during stimulus familiarization

Kenji W. Koyano *et al.*

Corresponding author: Kenji W. Koyano, kenji.koyano@nih.gov; David A. Leopold, leopoldd@mail.nih.gov

Sci. Adv. **9**, eade4648 (2023)
DOI: 10.1126/sciadv.ade4648

This PDF file includes:

Figs. S1 to S27

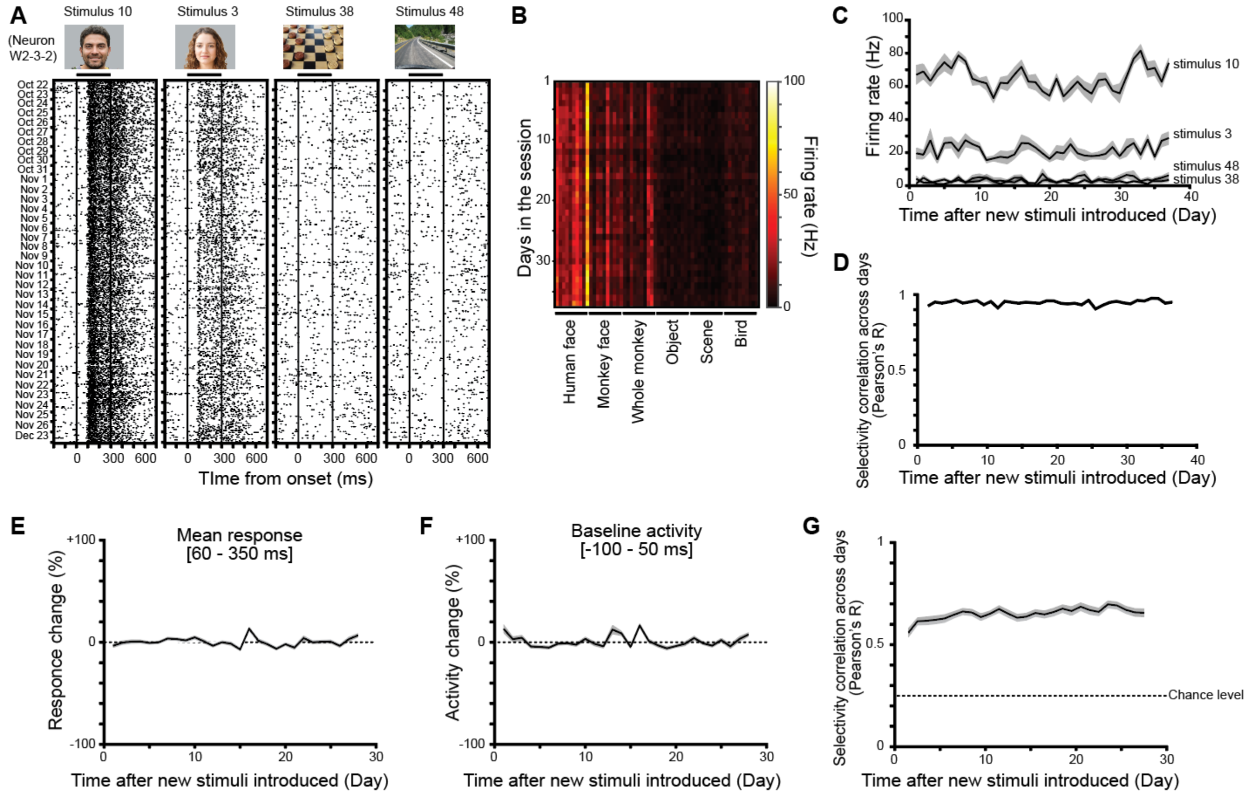

Figure S1. Stable response to familiar stimuli across days. **A**, Responses of an example neuron (neuron W2-3-2) to the familiar stimuli, which is consistent over the recording period of a month. **B**, Response of the neurons in **A** to all the 60 familiar stimuli, whose response pattern was preserved over the recording period of 37 days. **C**, Firing rate response of the neuron shown in **A** to some of the familiar stimuli over the recording period. **D**, Correlation of the response pattern of the neuron shown in **A**, between two consecutive recording days. **E**, Population mean response to the familiar stimulus set. Ordinate indicates the response deviation from the mean, which is stable across days. There was no significant difference between first and last two days (p=0.34, t_{135}=0.95, paired t-test). **F**, Population mean firing rate during baseline period, which is stable across days. There was no significant difference between first and last two days (p=0.07, t_{138}=1.84, paired t-test). **G**, Population average of correlation of the response pattern, which is well above the chance level during the recording session There was no significant difference between first and last two days (p=0.27, paired t-test, t_{93}=1.11). N=139 for **E-F.** N=94 for **G**, which does not include session W-1 where familiar stimuli were presented only on the first and last day of recording. Due to privacy right reasons, we display mock stimulus images for human faces in **A**, which were generated by artificial intelligence (https://this-person-does-not-exist.com/en).

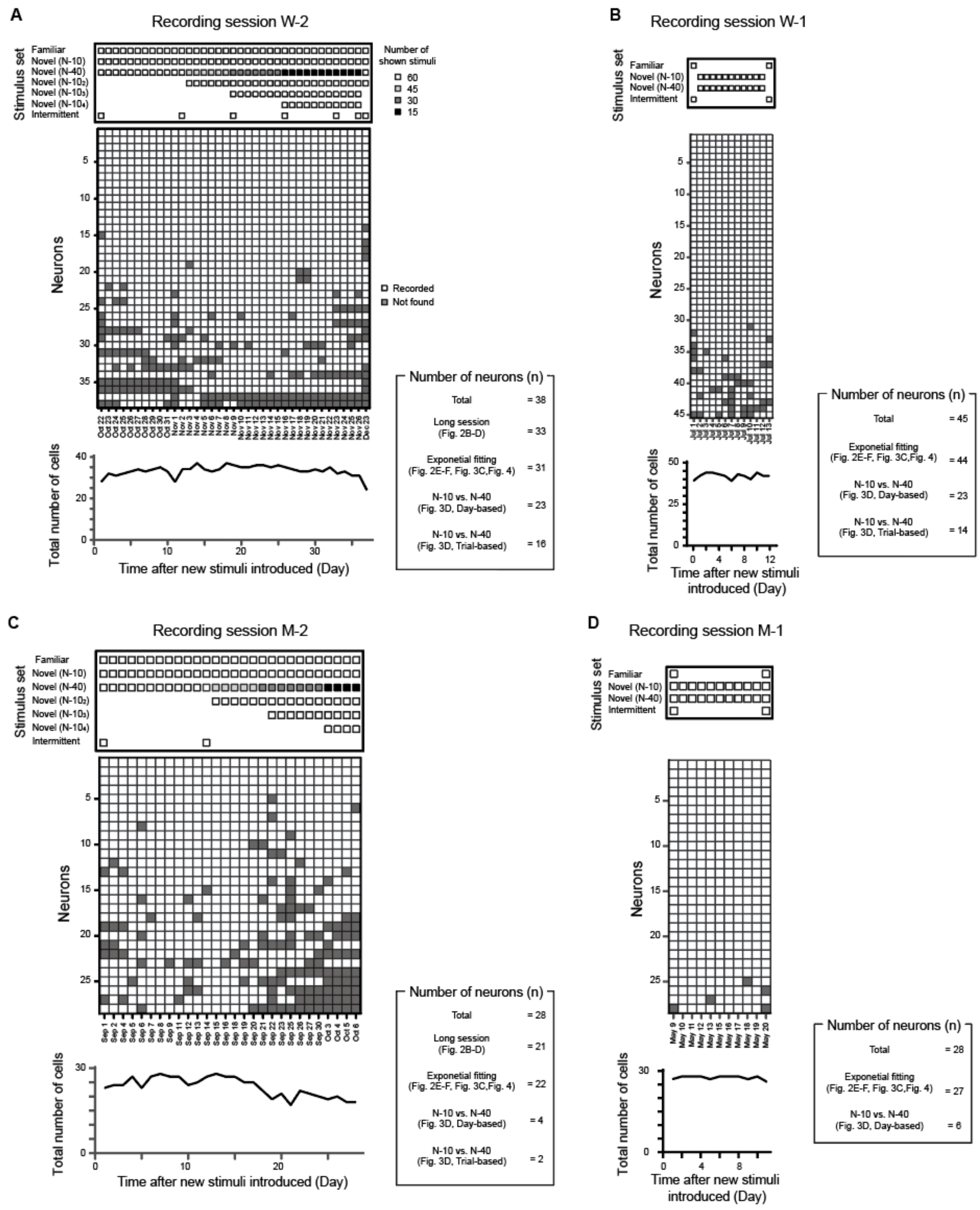

Figure S2. Schedule of stimulus presentation and recorded neurons for each recording session. **A**, recording session W-2. **B**, session W-1. **C**, session M-2. **D**, session M-1. Top, stimulus presentation schedule. Middle, recorded neurons on each day. Bottom, total number of neurons recorded on each day. Box on the right depicted the number of neurons used for each analysis. Session W-1 and W-2 were performed with animal W, and session M-1 and M-2 were performed with animal M. The same stimulus set was used for session W-1 and M-1, and another same stimulus set was used for session W-2 and M-1.

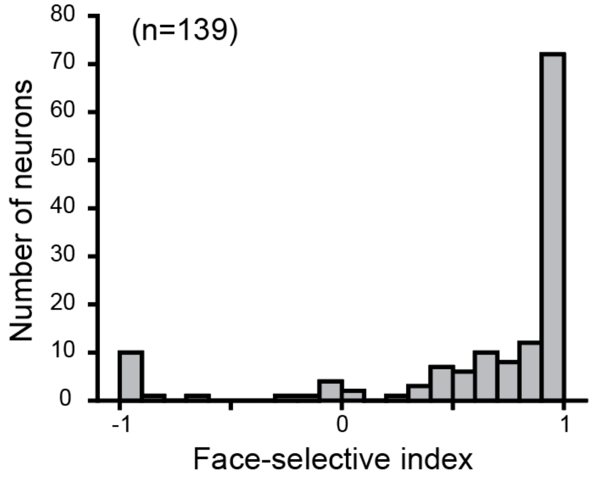

Figure S3. Distribution of face-selective index.

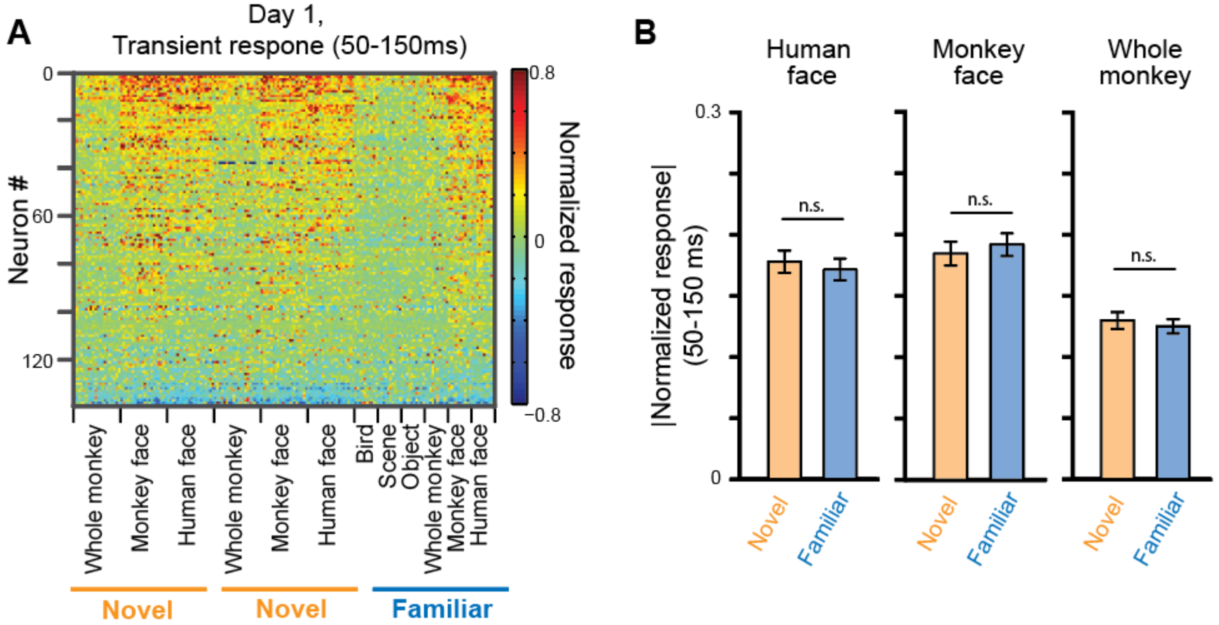

Figure S4. Early transient response on the first day. A and **B** are same as **Fig. 1F** and **H**, but for earlier transient response during 50-150 ms.

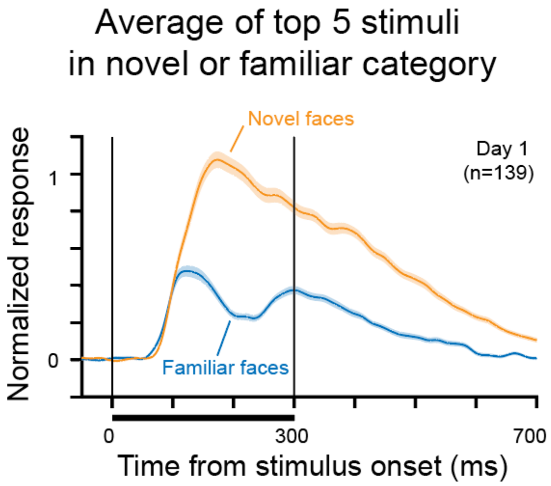

Figure S5. Time course of responses to novel and familiar face stimuli on the first day. Same as Fig. 1G but calculated from top-5 stimuli of each neuron. Shaded area indicates standard error from mean (S.E.).

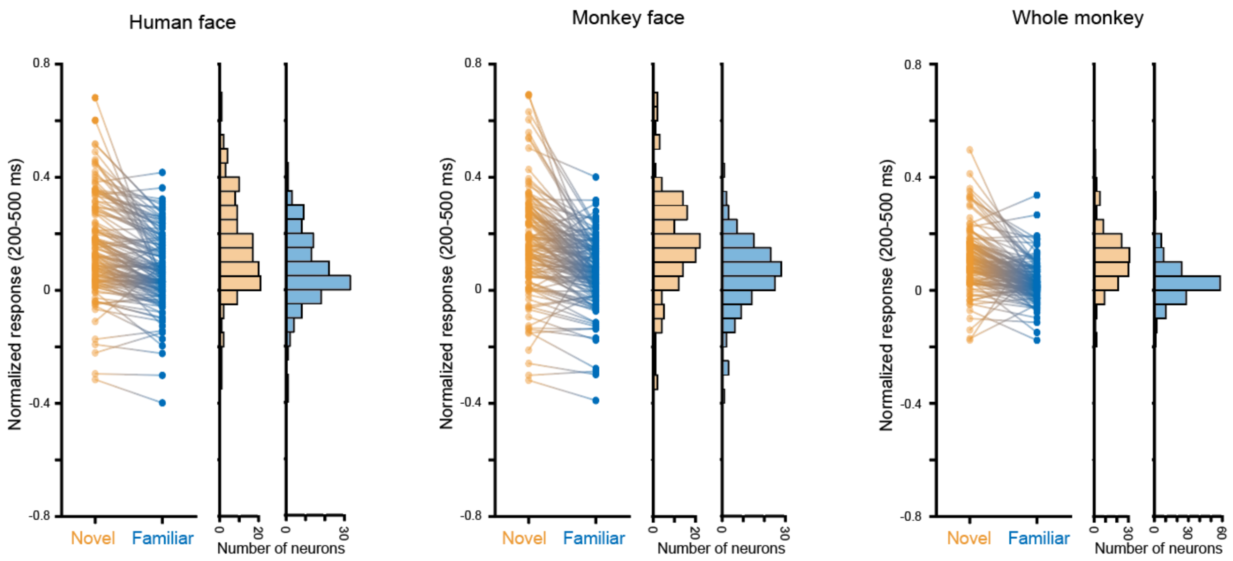

Figure S6. Average response to each category of stimulus on the first day during the late sustained response. Same as Fig. 1H but showing value of individual neurons and their distributions.

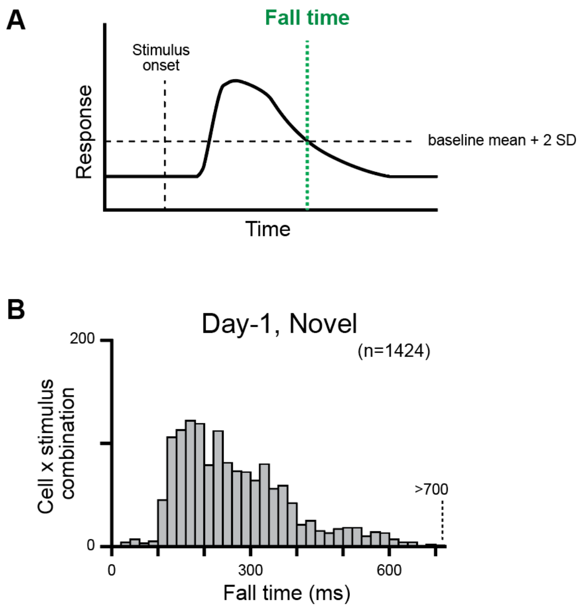

Figure S7. The duration of late sustained response. A, quantification of fall time, which is defined as the time from stimulus onset when the elevated response drops below the threshold line of baseline + 2SD, after exceeding another threshold line of baseline + 3SD at least once. **B,** Distribution of the fall time of responses to the novel stimuli on day 1. Of 1424 cases which evoked a response greater than 3SD, 481 (33.8%) showed fall time longer than stimulus presentation (300 ms).

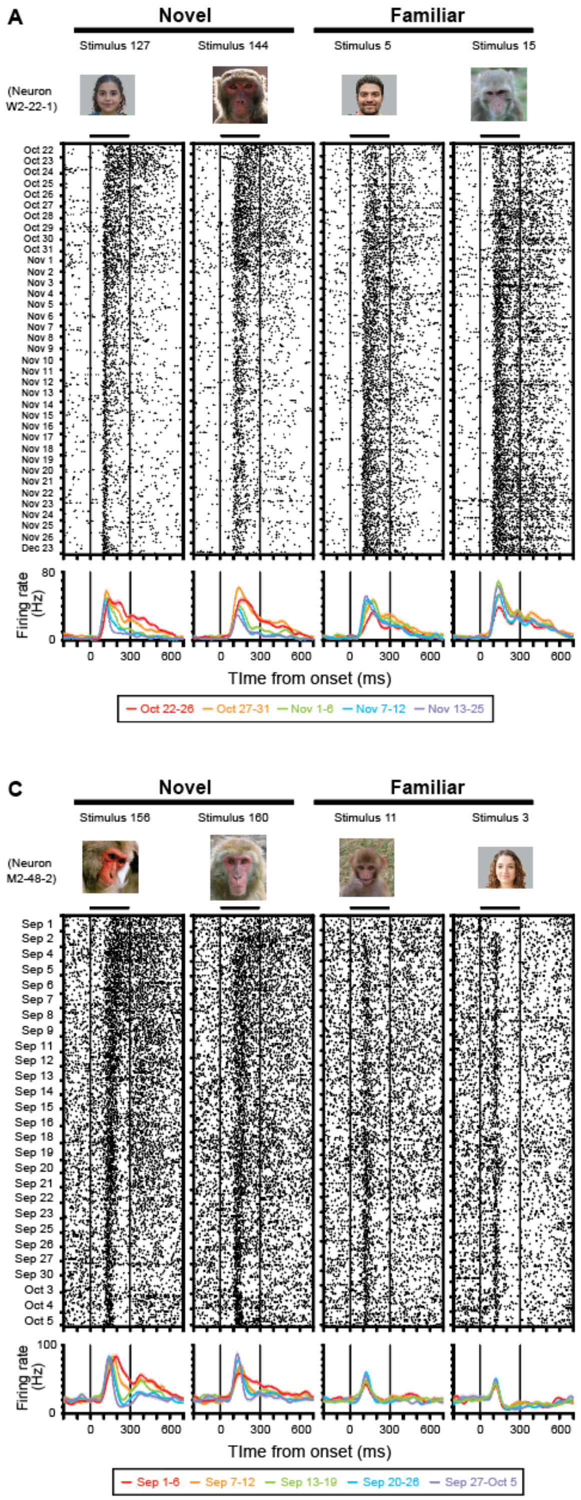

Figure S8. Additional examples of recorded neurons. The neurons showed response decrease in later sustained period for novel stimuli but not for familiar stimuli. Due to privacy right reasons, we display mock stimulus images for human faces, which were generated by artificial intelligence (https://this-person-does-not-exist.com/en). (Figure continues to the next page)

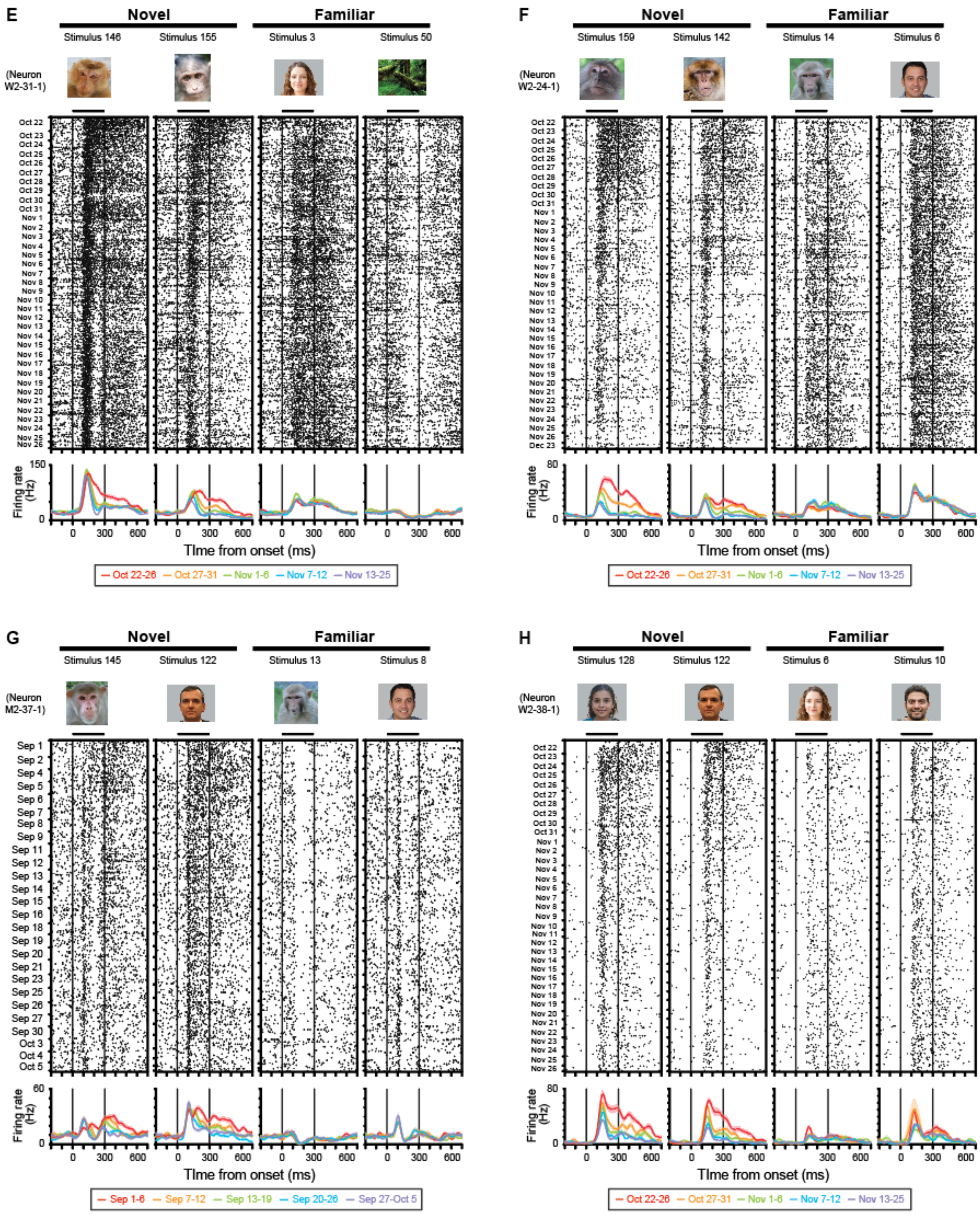

Figure S8 (continued)

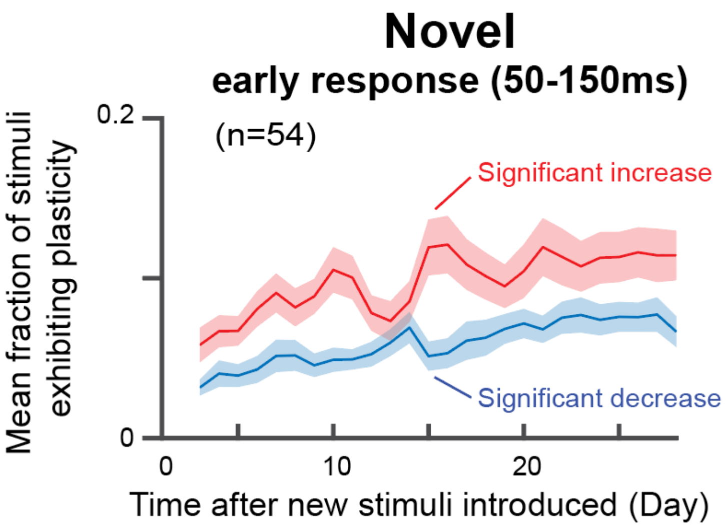

Figure S9. Mean fraction of stimuli which showed significant change of early transient response from that on first two days (*t*-test, *p*<0.05). Details are same as **Fig. 2D** but for earlier transient response during 50 to 150 ms after stimulus onset. There was a tendency of slight increase over time in the number of stimuli which showed significant increase.

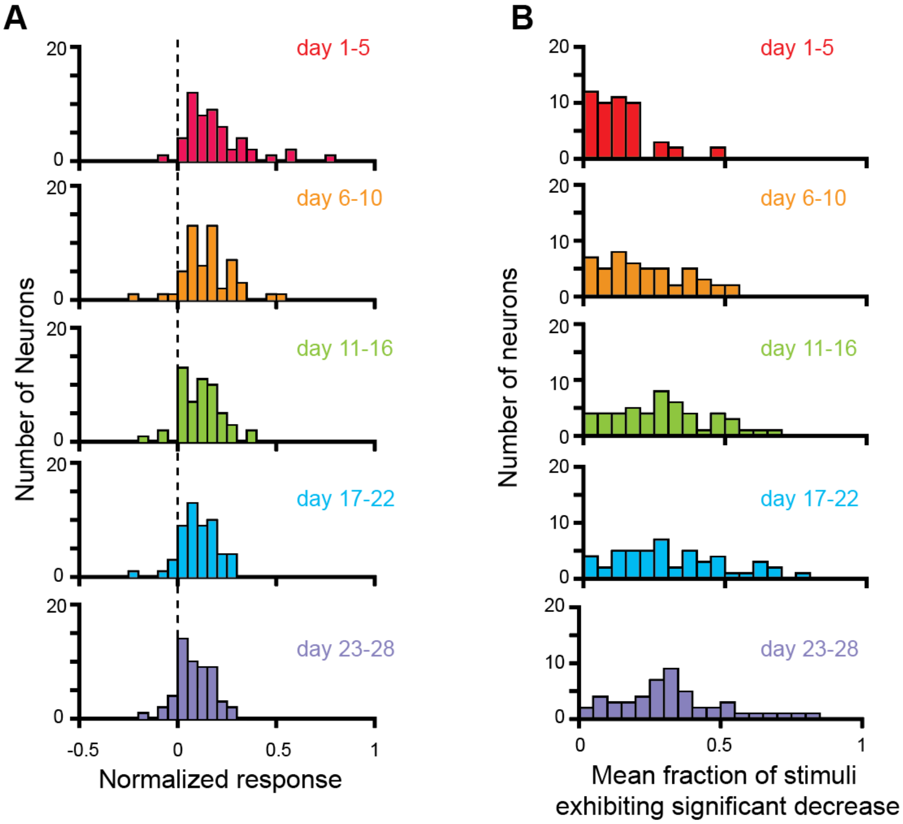

Figure S10. Distribution of late sustained responses over multiple days of visual exposure to initially novel stimuli. The same data in Fig 2C, D are plotted in different formats. **A**, Distribution of response during late sustained period (200-500 ms after stimulus onset) over four weeks. **B**, Distribution of mean fraction of stimuli showing significant population response decrease from the first two days (*t*-test, $p<0.05$).

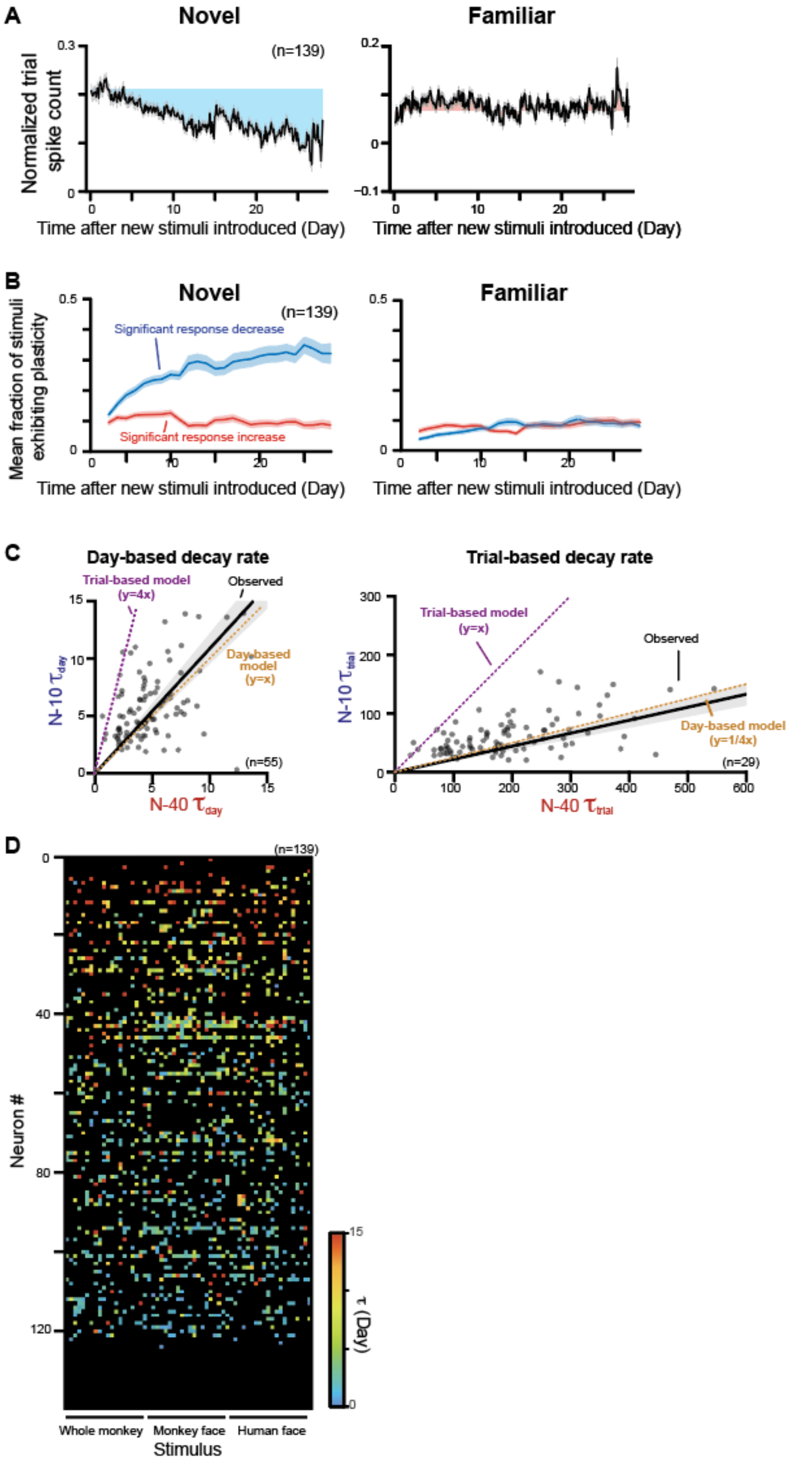

Figure S11. Response change with repeated exposure to novel stimuli. Same as in Fig. 2C, D, Fig 3D and Fig.4B but calculated with all the neurons (n=139). Decrease of response, day-based decay and dependence of time constant is replicated as Fig. 2-4. Note that number of recorded neurons are different across days (see also Fig. S2).

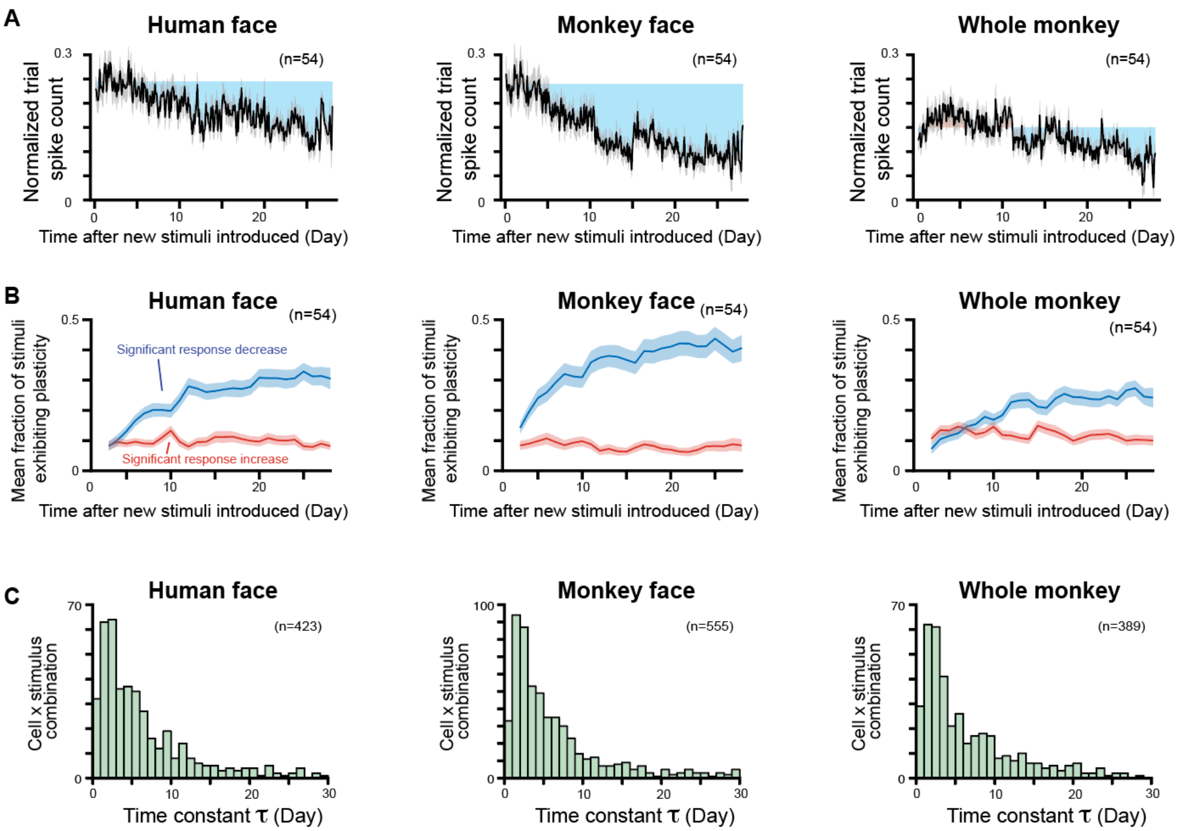

Figure S12. Decrease of sustained response with visual exposure to the three categories of the novel stimuli. A-C, Same as Fig. 2C, D, F but separately plotted for each stimulus categories of human face, monkey face and whole monkey. Response decay is larger for monkey face stimuli and larger fraction of monkey face stimuli exhibited plasticity. The distributions of time constant *tau* are similar across stimulus categories and not significantly different (p=0.88, Kruskal-Wallis test; median = 4.31, 4.16 and 4.10 for human face, monkey face and whole monkey, respectively).

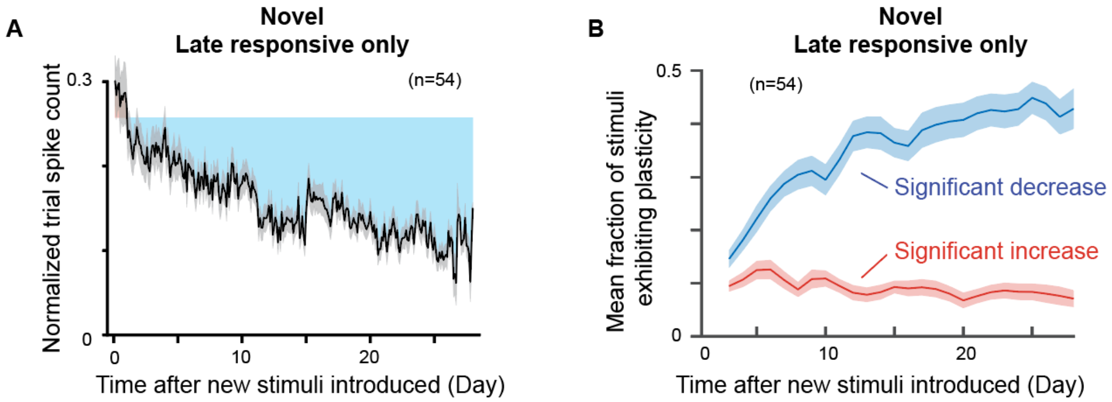

Figure S13. Change of responses to novel stimuli which elicited late sustained response. A and **B** are same as **Fig. 2C** and **D** but calculated only for the stimuli which showed significantly higher late sustained response than baseline (p<0.05, *t*-test with Bonferroni correction).

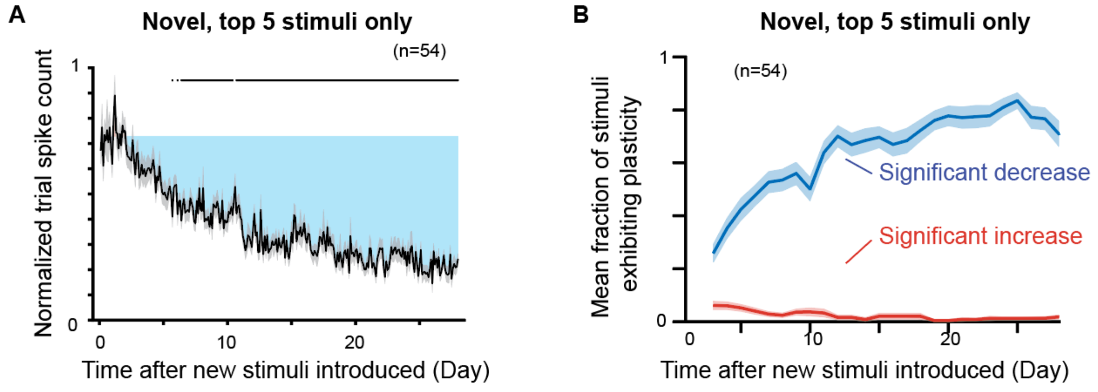

Figure S14. Change of responses to top-5 novel stimuli. A and **B** are same as **Fig. 2C, D** but calculated only for the stimuli which elicit first to fifth largest late sustained response.

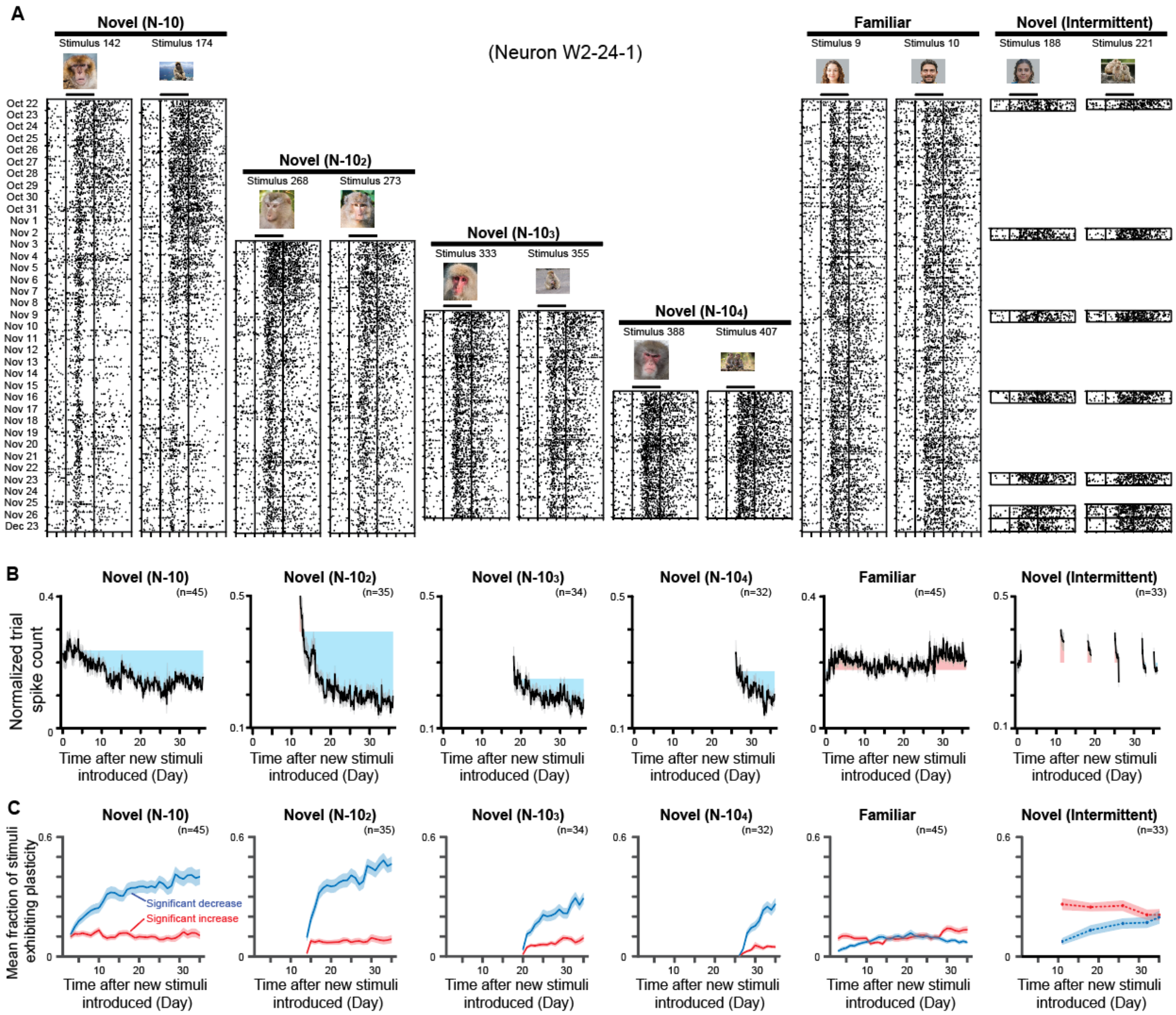

Figure S15. Response decreases for newly introduced novel stimuli at the middle of the recording session. **A**, example response of a neuron (Neuron W2-24-1). **B**, Change of response during late sustained period over four weeks. **C**, Mean fraction of stimuli which showed significant change from first two days (*t*-test, $p<0.05$). **B** and **C** included neurons which was isolated at least 70% of the first 36 days (26 days) of recording. Details of **B** and **C** are same as **Fig. 2C** and **D**. Due to privacy right reasons, we display mock stimulus images for human faces, which were generated by artificial intelligence (https://this-person-does-not-exist.com/en).

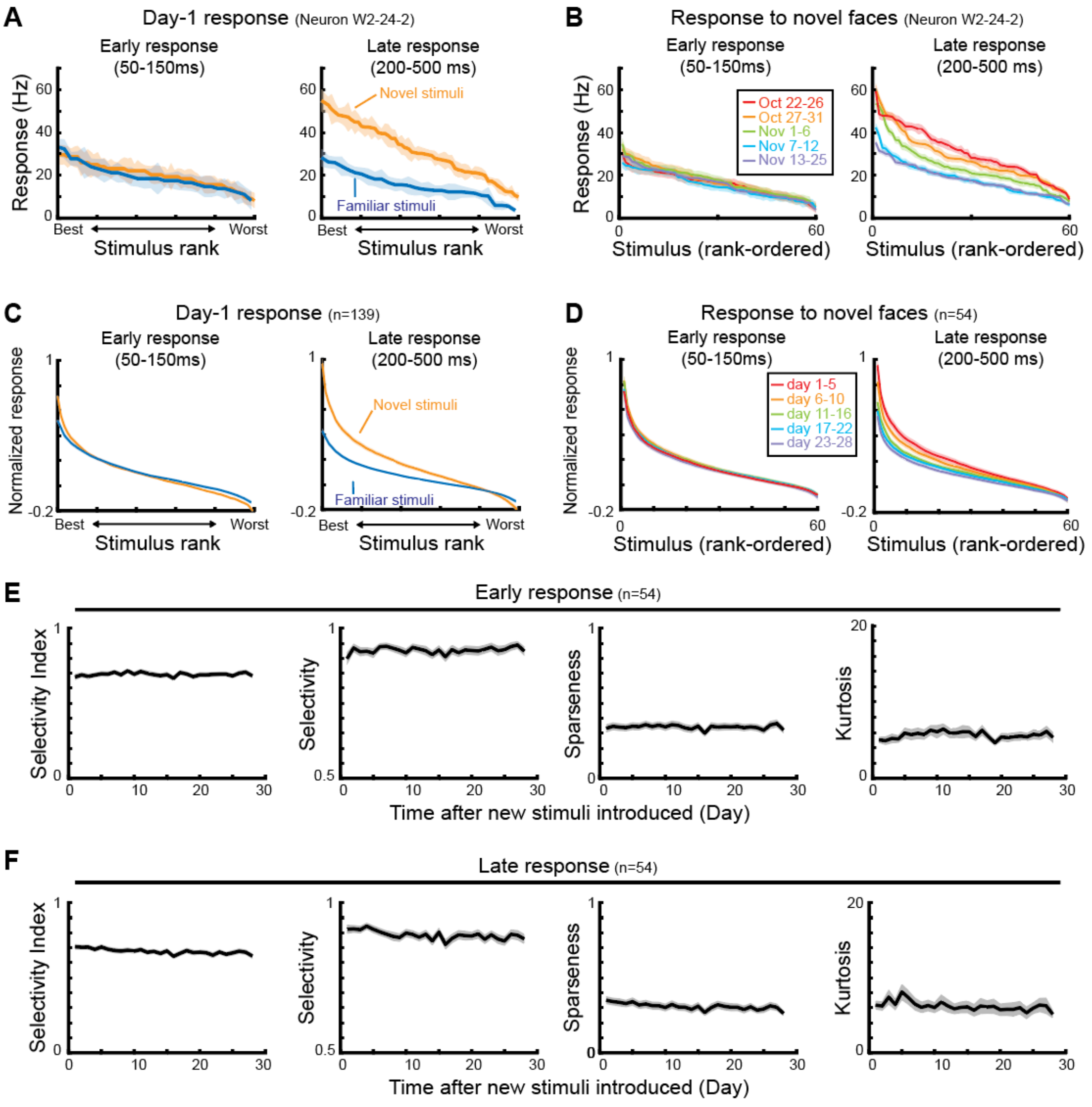

Figure S16. Response change with constant stimulus selectivity. A, Response of an example neurons to novel and familiar stimuli. Stimuli are rank-ordered based on the magnitude of the response. **B**, Response change of the example neuron during visual exposure to initially novel stimuli. Stimuli are rank ordered for each day according to elicited responses. **C**, Population mean response to novel and familiar stimuli on the first day. Stimuli are rank ordered for each neuron. **D**, Population mean response to initially novel stimuli during visual exposure. Stimuli are rank ordered for each neuron and each day according to the responses elicited. **E**, Value of selectivity/tuning indices of early response for the initially novel stimuli during the visual exposure. From left to right, Selectivity index, Selectivity, Sparseness, and Kurtosis. See Methods for the detailed definition of the indices. **F**, same as **E**, but for late sustained response. Shaded area of each panel, S.E.

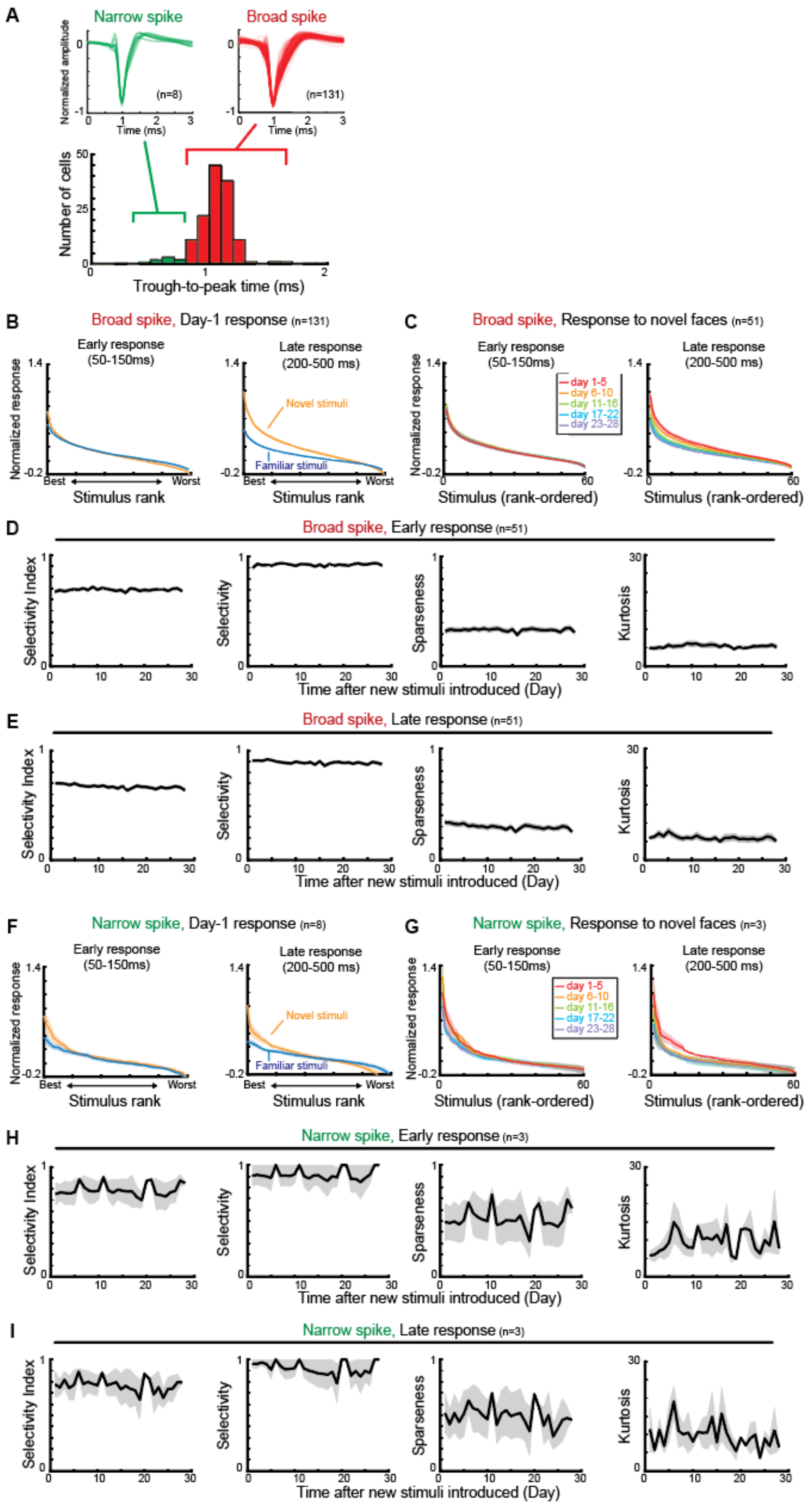

Figure S17. Response change with constant stimulus selectivity and tuning in both broad and narrow spike neurons. A, Distribution of trough-to-peak time width of spike waveform. Green, narrow spike waveform. Red, Broad spike waveform. Threshold between the two is 0.8 ms. **B-E**, same as Fig. S16C-F but for neurons with broad spike. **F-I**, same as Fig. S16C-F but for neurons with narrow spike.

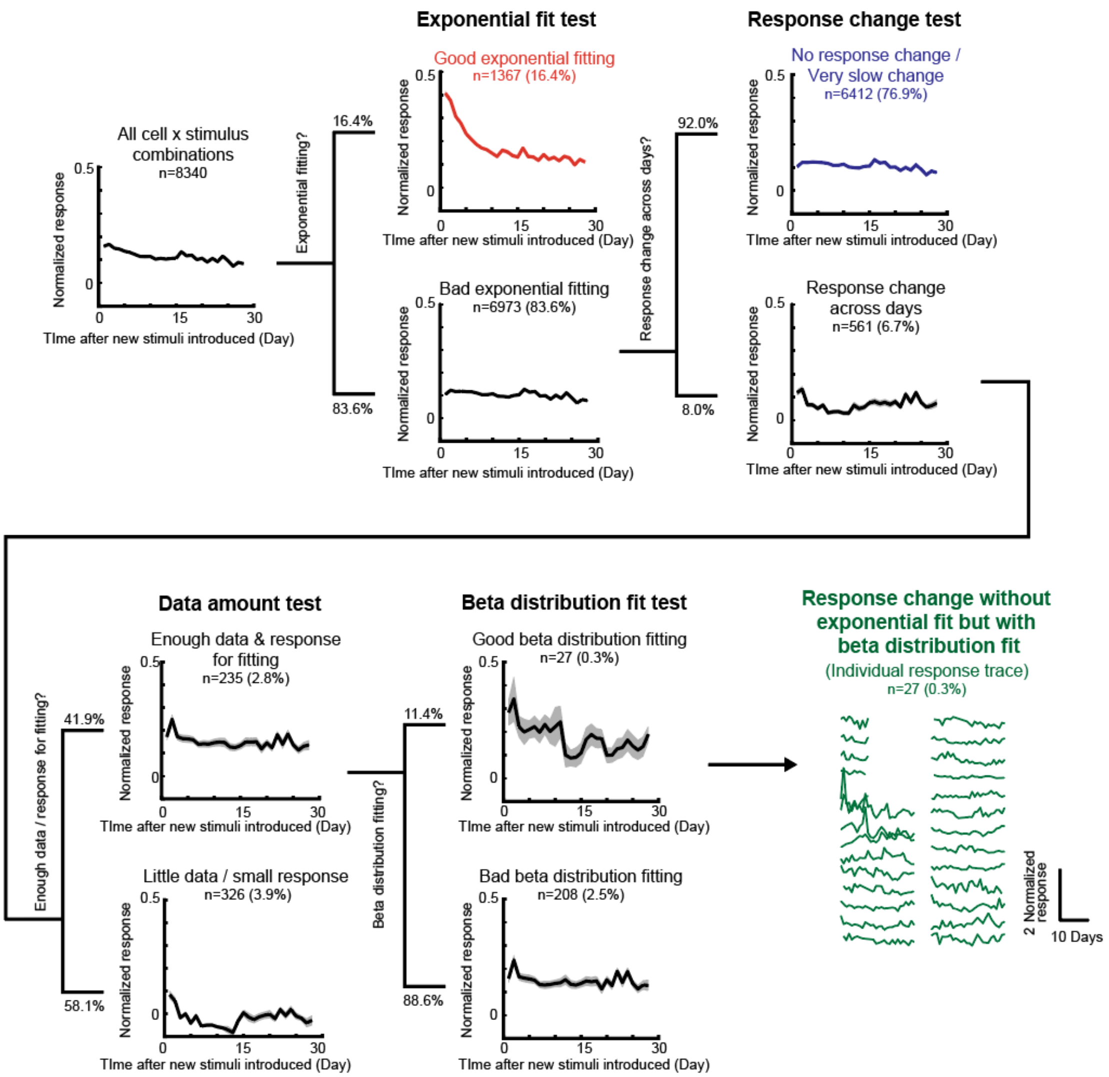

Figure S18. Response pattern over the visual exposure to the novel stimuli. The response patterns were classified with various steps of test. First, exponential fit test was performed by checking whether the response patterns fulfil the criteria for good exponential fit or not (see Methods for details of the criteria). Of all possible 8340 cell x stimulus combinations, 16.4% had good exponential fit (*red plot on the top*). Next, the 6973 response patterns without good exponential fitting were tested whether the response changes or not. Patterns without significant difference between first and last two days by *t*-test, or patterns with very slow time constant longer than 30 days, were classified as no response change / very slow change neurons (*blue plot on the top right*). With this test, 92.0 % of the bad exponential fitting (or 76.9% of all possible combinations) were classified into this category. Then, the 561 response-changing patterns were further tested for data amount test. With this test, 58.1% (or 3.9% of all possible combinations) of patterns, which showed mean responses less than 2Hz or were isolated for less than 7 days of the first 10 days, were classified as patterns with little data or small response. Finally, the response patterns of 235 cell x stimulus combinations, which cannot fit well with exponential function, change response across days and had enough amount of data, were tried to be fitted with beta function which had greater capacity to fit various types of timecourse than exponential fit. This successfully fit 11.4% cases (or 0.3% cases of all possible combinations) with various, non-consistent pattern (*green plot on the bottom right*). The other 88.6% (or 2.5% cases of all possible combinations) cannot be fit well even with beta function, probably because of their noisy response pattern across days.

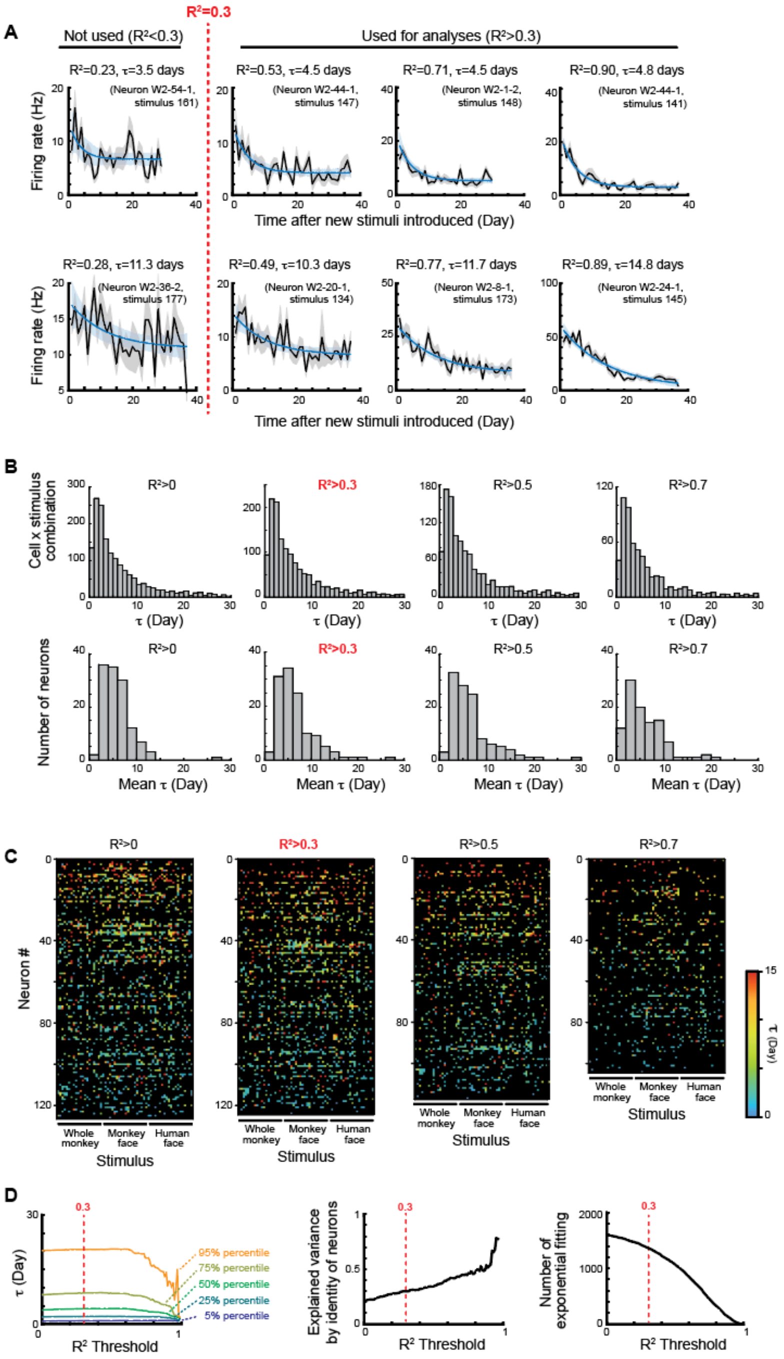

Figure S19. Effect of R^2 threshold of exponential fitting on the time constant. **A,** Example of exponential fitting. *Top*, fit with τ<5 days. *Bottom*, fit with τ>10 days. Plots are sorted with R^2 values, from left to right. Fit with $R^2 > 0.3$ was adopted in this study, together with a few more additional criteria for good exponential fitting (see Methods for details). **B**, Distribution of τ as Fig. 2F and 4C, but at different R^2 threshold values. **C**, Time constant for all cell x stimulus combinations across the population as Fig. 4B but at different R^2 threshold values. **D**, Quantification of the τ distribution (*left*), Explained variance by neuron identity (*middle*) and number of valid exponential fitting (*right*) at different R^2 threshold values. Note that other criteria for good exponential fit, like significant difference between first and last two days, were also applied in addition to the R^2 thresholding. Explained variance was calculated from R^2 statistic of one-way ANOVA.

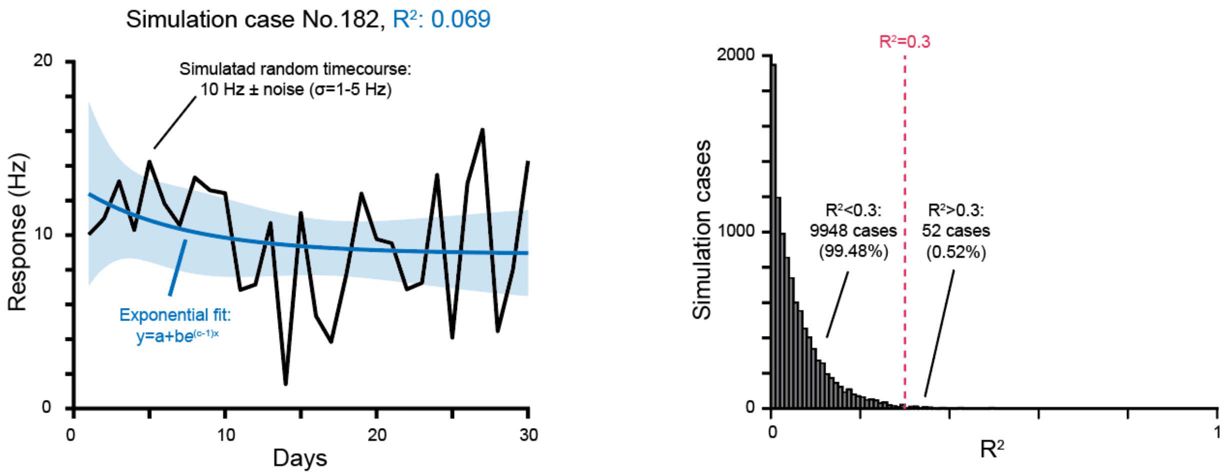

Figure S20. Simulation for evaluation of R^2 value of the exponential fitting using random time course. *Left*, an example fitting with the exponential function. The random time course is generated for 30 days as a combination of 10 Hz constant and gaussian noise. The range of standard deviations of the gaussian noise, 1-5Hz, is randomly determined for each time course pattern. Shaded area is 95% confidence interval of the exponential fit. *Right*, distribution of R^2 values with 10,000 repetitions of the exponential fitting stimulation. With threshold of R^2=0.3, 99.48% of cases are rejected and only 0.52% cases of random time course fulfill the criteria of R^2 value.

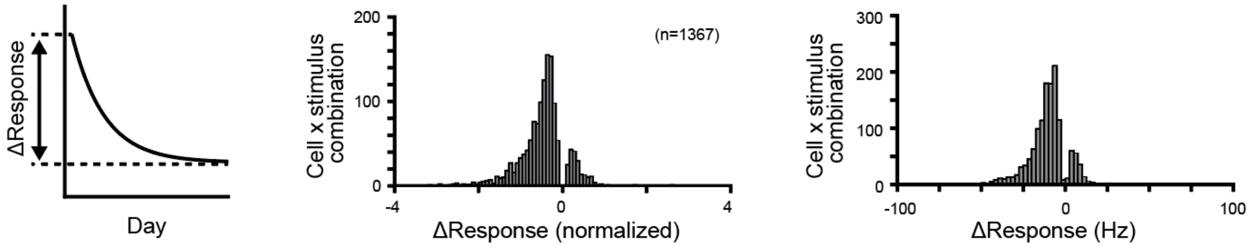

Figure S21. Magnitude of the late response change to the novel stimuli. *Left*, measurement of response change (ΔResponse) from the exponential fitting. *Middle*, distribution of ΔResponse. Responses are normalized for each neuron. The population response is decreasing (-0.46 ± 1.83, $p<10^{-19}$). *Right*, distribution of ΔResponse without normalization. The population response is decreasing (-12.6 ± 23.4 Hz, $p<10^{-77}$). Of the 1367 neuron x stimulus combination of exponential fit, 1170 (85.6%) are decreasing (median change: 11.9 Hz) and 197 (14.4%) are increasing (median change: 7.5 Hz).

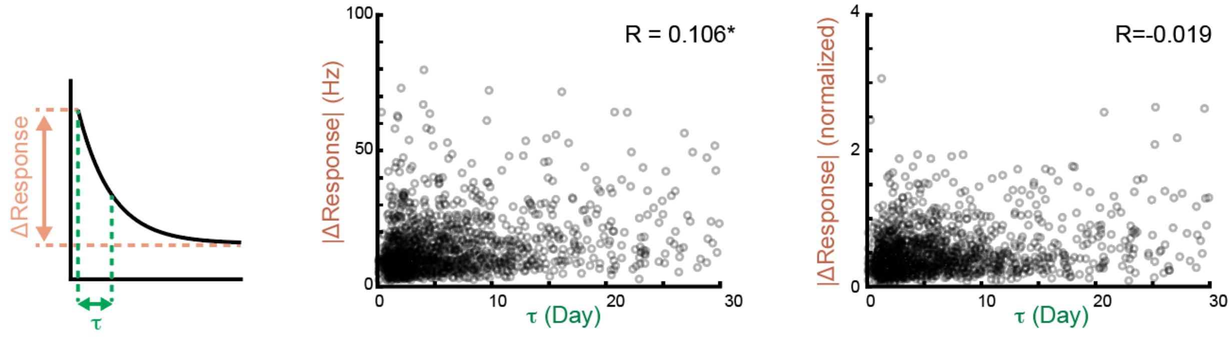

Figure S22. Relationship between the magnitude of the change and time constant of the change. *Left*, measurement of the change of the response (ΔResponse) and the time constant (τ). *Middle* and *Right*, scatter plot showing relationship between τ and the absolute value of ΔResponse. Response is normalized for each neuron on the right plot. *, p<0.001.

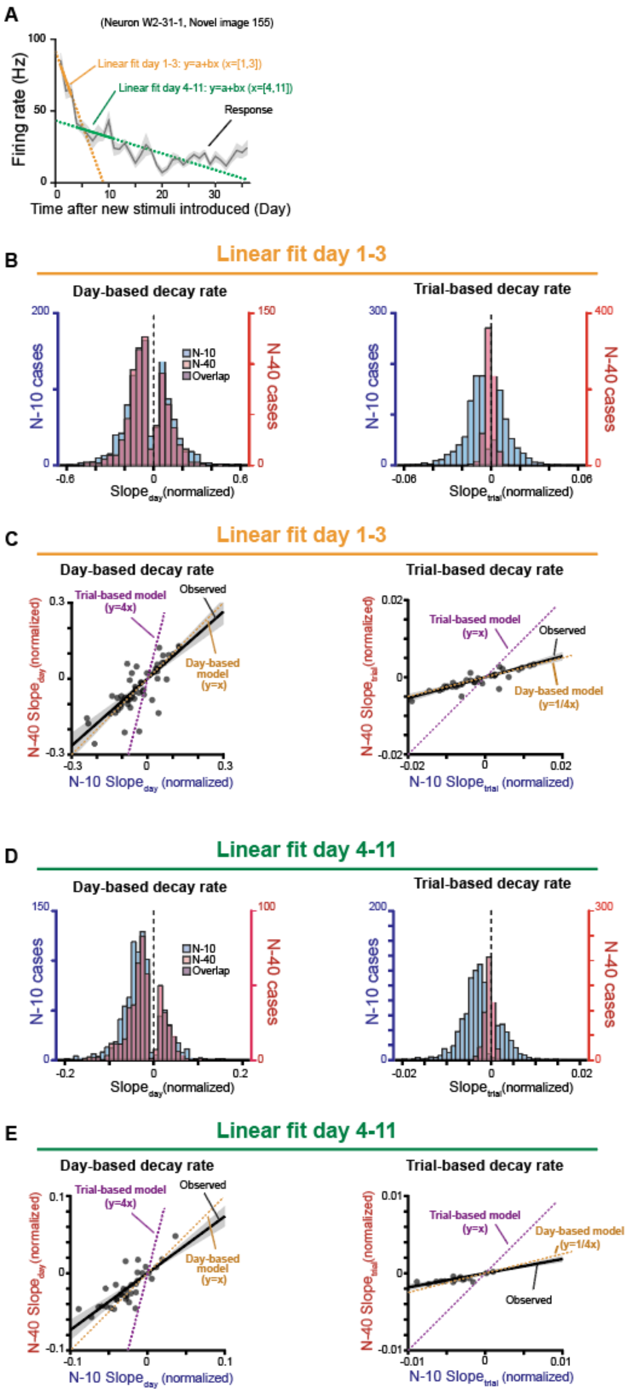

Figure S23. Primary factor of day on the rate of visual response plasticity at the beginning (days 1-3) and subsequent (days 4-11) periods of visual exposure. A, An example of linear fitting to measure decay rates as the slope of the fitting for the first 3 days (orange) and following 8 days (green). **B,** Population distribution of estimated fitting slope for the first 3 days showing large overlap of day-based slope, which indicate importance of day as a critical factor. Same as Fig. 3C but for slopes of the linear fit. **C,** Within-cell comparison between the slopes of N-10 and N-40 stimuli for the first 3 days. Same as Fig. 3D but for slopes of the linear fit. The observed slopes (black) follow day-based model (brown). **D** and **E**, same as **B** and **C** but for days 4-11. Similarly to days 1-3, day is a critical factor for days 4-11.

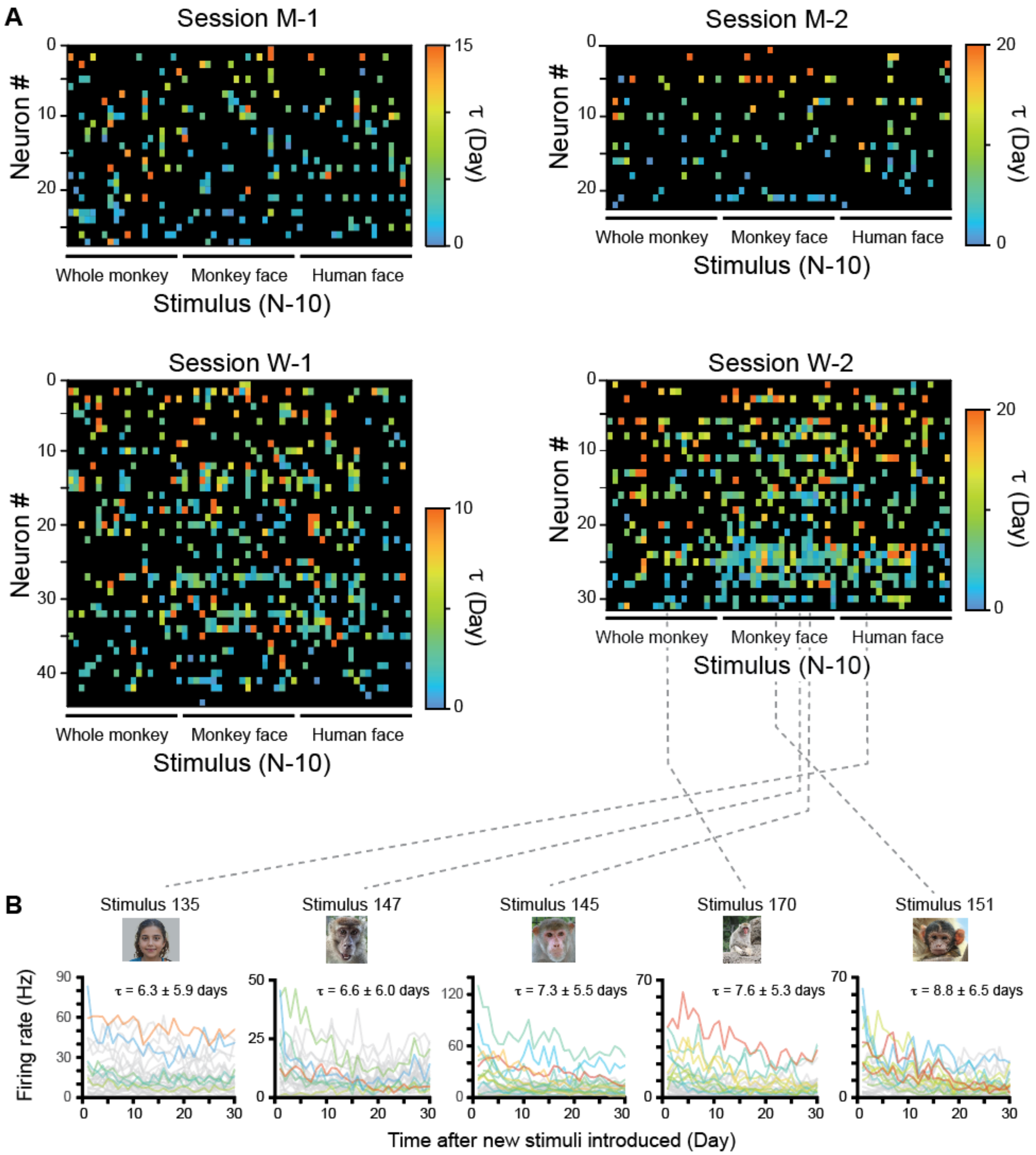

Figure S24. *Tau* for each recording session. A, Time constant for all cell x stimulus combinations of each session. Details are same as **Fig. 4B** but plotted for each recording session. Main effect of cell: session M-1, $p<0.01$; session M-2, $p<0.0001$; session W-1, $p<0.03$; Session W-2, $p<10^{-8}$ (Kruskal-Wallis test). Main effect of stimuli: session M-1, $p=0.46$; session M-2, $p=0.68$; session W-1, $p<0.04$; Session W-2, $p<10^{-5}$ (Kruskal-Wallis test). **B**, Example stimuli of session W-2, showing large variety of plasticity time constants for each stimulus. Details are same as **Fig. 4A** but plotted for each stimulus. Each trace represents response time series of each neuron for the stimulus (n=31 neurons per plot). Due to privacy right reasons, we display mock stimulus images for human faces in **B**, which were generated by artificial intelligence (https://this-person-does-not-exist.com/en).

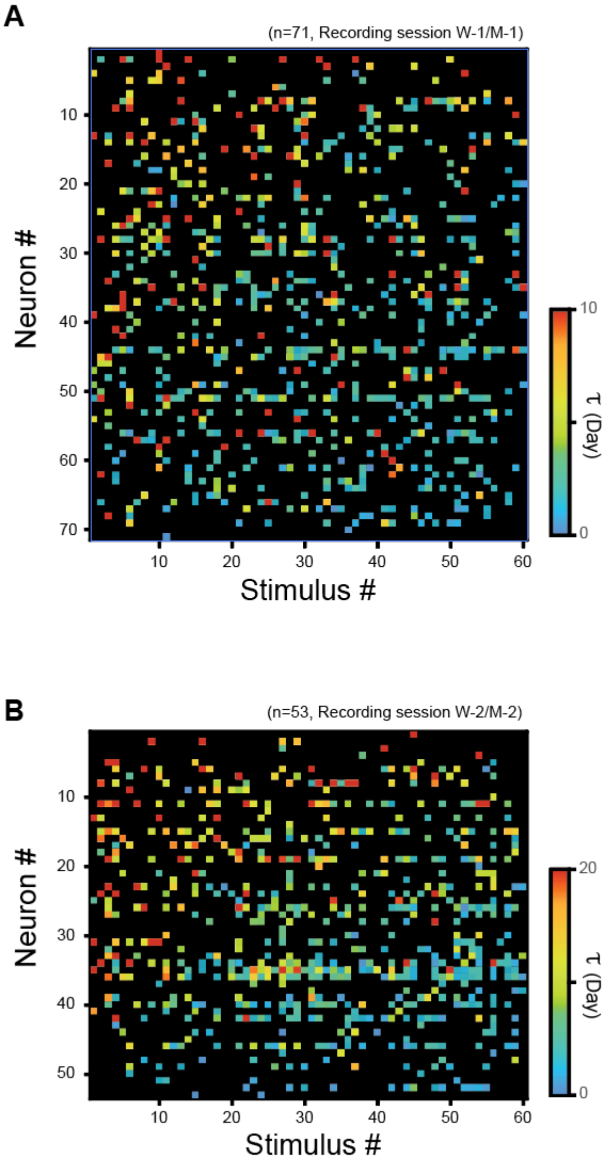

Figure S25. *Tau* for two group of recording sessions which are using the same stimulus set. **A** and **B**, Time constant for all cell x stimulus combinations of each session. Details are same as **Fig. 4B** but plotted for recording session W-1 and M-1 (**A**) and W-2 and M-2 (**B**). Stimuli are sorted according to average τ value. Main effect of cell: session M-1/W-1, $p<0.0005$, explained variance=0.15; session M-2/W-2, $p<10^{-16}$, explained variance=0.25 (two-way ANOVA after log transformation of τ). Main effect of stimulus identity: session M-1/W-1, $p=0.20$, explained variance=0.085; session M-2/W-2, $p<0.0001$, explained variance=0.13 (two-way ANOVA after log transformation of τ).

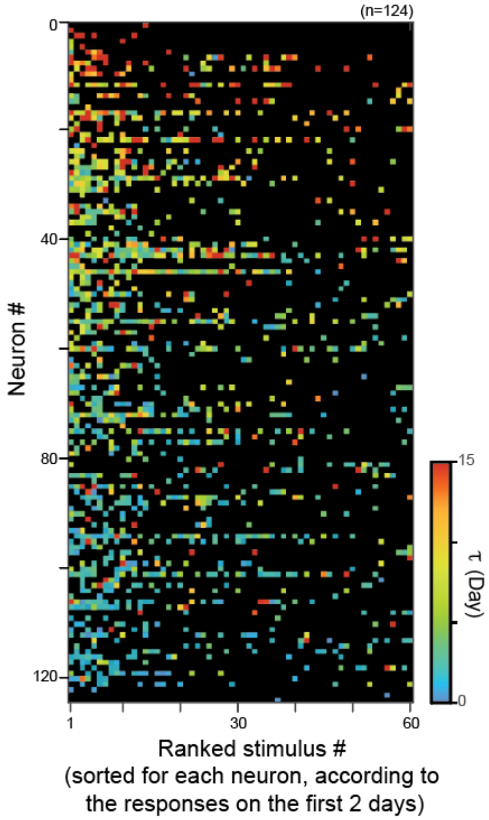

Figure S26. Time constant of plasticity for all cell x stimulus combinations. Same as Fig. 4B, but stimuli are sorted for each neuron according to the responses on the first two days. Stimuli with higher rank tended to exhibit response change which meet criteria of the exponential fit, but there is no clear relationship between the stimulus rank and time constant τ. Two-way ANOVA after log transformation of τ showed significant main effect of neuronal identity ($p<10^{-26}$) but no significant main effect of stimulus rank ($p=0.22$) on the time constant τ.

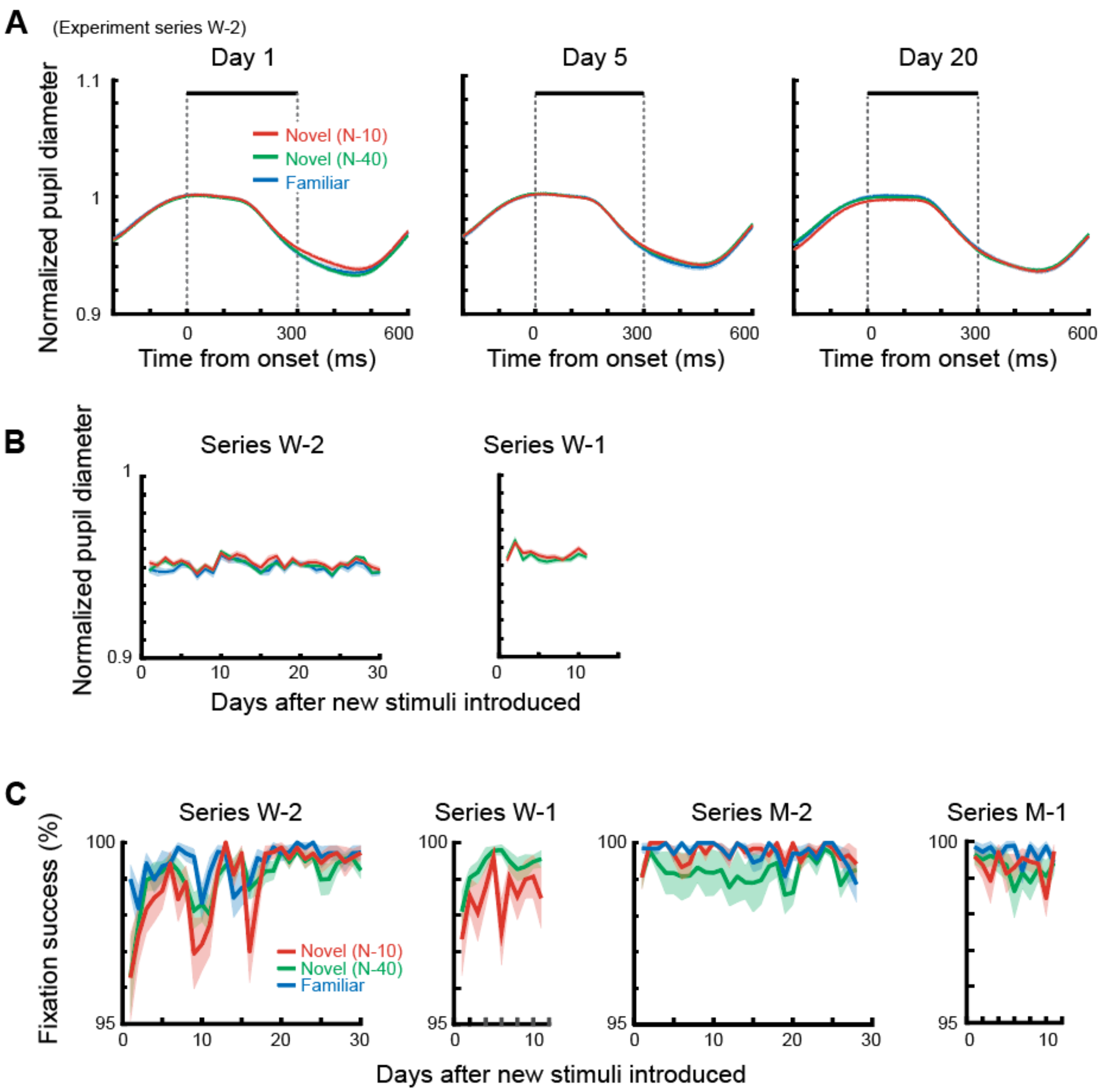

Figure S27. Response of the animals during the sequential passive viewing task. A, Pupillometry timecourse on day 1, 5, and 20 of experimental series W-2. There are no clear differences between familiar and novel stimuli and no clear changes across days. **B**, Stable pupil diameter across days. Pupil diameter during 200-500 ms after the stimuli onset was averaged for the novel and familiar stimuli. There was no difference of the pupil diameter between novel and familiar stimuli (p=0.12-1, t-test with Bonferroni correction), except for day 3 (p<0.03, t-test with Bonferroni correction). **C**, Fixation performance in each experimental series. Animal W (series W-1 and W-2) exhibited improvement of fixation success rate over days, while animal M (series M-1 and M-2) showed no clear change in the trend of fixation success rate. Shaded area, S.E.
